# Lipid metabolism, microglia, and stroke

**DOI:** 10.4103/NRR.NRR-D-24-01523

**Published:** 2025-08-13

**Authors:** Lei Chen, Minmin Zhang, Wei Wei, Qiang Li, Lijun Wang, Ming Zhao, He Li, Hongye Xu, Pengfei Yang, Ping Zhang

**Affiliations:** 1 Department of Neurovascular Center, Changhai Hospital, Naval Medical University, Shanghai, China; 2 Department of Neurology, Naval Medical Center of PLA, Naval Medical University, Shanghai, China; 3 Department of Emergency, Naval Medical Center of PLA, Naval Medical University, Shanghai, China

**Keywords:** cerebral hemorrhage, diet, gut microbiome, inflammation, ischemic stroke, lipid, metabolism, microglia, regeneration, therapeutic targets

## Abstract

Microglia, lipids, and their interaction are found to play important roles in post-stroke immunity. Microglia are sensitive to detect environment change in injured brain. Activated microglia undergo phenotypical remodeling and trigger complex signal cascades to regulate immune responses after stroke. Lipids including peripheral lipid metabolism and lipid droplet biogenesis are involved in the control of microglia functions, such as activation, phagocytosis, proliferation, and pro-inflammation. In this review, we explore new scope of microglia and lipids in immune regulation of stroke. Implication of peripheral lipid metabolism after stroke is mentioned and advances in microglia-lipid interaction are discussed. We give a special focus on how diet and gut microbiome influence neuroinflammation system via gut–brain axis, and how these processes associate with the risk and outcome of stroke. Moreover, we reviewed the therapeutic targets related to lipid metabolism and microglial modulation after stroke. These can provide a prospective strategy for more efficient and safer treatment for ischemic and hemorrhagic stroke.

## Introduction

Stroke is classified as ischemic and hemorrhagic, and 62.4% of all incident strokes are ischemic (GBD 2019 Stroke Collaborators, 2021). Ischemic stroke occurs due to insufficient blood supply to the brain, resulting in lack of oxygen and energy that leads to acute brain harm. Intracerebral hemorrhage is uniquely devastating with a high mortality rate (Xue and Yong, 2020). The initial hemorrhage and hematoma expansion caused mechanical disruption constitute the primary injury. A secondary injury which is driven by the release of ferrous iron, edema, and neuron excitotoxicity soon develops after hemorrhage (Tschoe et al., 2020).

The pathological process of stroke involves a series of responses, such as microglia activation, inflammation, oxidative stress, and peripheral immune cells recruitment evolves over hours to days and even weeks (Kim et al., 2019b; Bai et al., 2020; Tschoe et al., 2020). In response to acute insults, complex immune cascades take place and cytokines, chemokines and other modulatory molecules are produced, promoting or attenuating brain injury, and participating in subsequent brain recovery. After stroke, microglia are rapidly activated before neuronal cell death and play an important role in innate immunity (Iadecola et al., 2020; Li et al., 2020b). Then the developing neuroinflammation causes additional injury to the brain, resulting in cell death in the acute phase, but conversely plays a beneficial role in repair activities and promotes recovery in post-stroke repair phase (Campbell and Khatri, 2020; Mo et al., 2020). Brain tissue repair is facilitated attributed to neurotrophic factors secreting and debris clearing, which is mainly mediated by microglia and lipid metabolism (Leyrolle et al., 2019; DeLong et al., 2022).

Lipids are dominant composition of cerebral tissue and are essential for sustaining normal function and homeostasis of central nervous system. Various lipids such as simple lipids, phospholipids, glycolipids and fatty acids are also major components of the cellular membrane (Andreone et al., 2017). The homeostatic essential lipids metabolism is impaired in cerebral ischemia because of the severe shortage of cerebral blood flow (Paik et al., 2009; Shanta et al., 2012). Various lipid mediators produced after stroke participate in the regulation of both the pro-inflammatory and the pro-resolution process in ischemic injury (Nakamura et al., 2020). A large amount of evidence demonstrated that high levels of plasma cholesterols are associated with a larger infarction size and a higher extent of edema (Kim et al., 2008, 2020b; Navi and Segal, 2009; ElAli et al., 2011). The damage induced by hyperlipidemia is closely related to the increased pro-inflammatory reactions within the injury lesion after stroke (Kim et al., 2012).

Besides acute reperfusion therapy, no other treatments can improve ischemic stroke outcomes efficiently so far. However, only 27% of ischemic stroke patients can be free of disability 3 months after reperfusion therapies (Renú et al., 2022). The available therapy regimens are even less and prognoses are even poor in intracerebral hemorrhage. Understanding the inflammation process after stroke may be helpful in finding innovative and effective treatment targets that can mitigate or even inhibit brain injury. This review primarily focuses on lipids and microglia involved immune process after stroke. Then, it also states how diet and gut microbiome influence stroke and discusses the development of new therapeutic strategies for stroke.

## Search Strategy

The PubMed database was searched using the following search terms: “lipids,” “lipid metabolism,” “lipidomics,” “microglia,” “phenotype,” “inflammation,” “diet,” “gut microbiota,” “ischemic stroke,” “cerebral hemorrhage,” “therapy,” and “treatment.” All results were further screened by title and abstract, including only articles presenting microglia and lipids mediated function in stroke, focusing on their interaction in immune regulation and targeted treatment strategy, from 2001 to 2025. The conference abstracts, case reports, and studies only for chemical analysis of lipids without their function on brain tissue were excluded.

## Lipid Metabolism and Transport

The role of lipids in physiological functions is profoundly significant. Cholesterol not only contributes to the stabilization of cell membranes but also facilitates transport across these membranes (Huang et al., 2025). Additionally, it is vital for hormone synthesis, acting as a precursor for hormones associated with the adrenal glands and sex hormones. Furthermore, cholesterol is essential for bile acid synthesis, which plays a crucial role in the fat absorption process within the small intestine (Woo et al., 2022). Triglycerides (TG), on the other hand, serve as an effective form of energy storage and production. The glycogen density in the liver or muscle is higher than the TGs in adipose tissue (Inoue et al., 2022). The heart and skeletal muscles utilize circulating TGs, converting them into fatty acids and glycerol through lipolysis. Fatty acids act as the primary energy source for muscle cells, undergoing breakdown in oxidation reactions before entering the Krebs cycle, ultimately leading to the production of adenosine triphosphate (Schmidt et al., 2020).

### Lipoprotein complexes

The primary lipid particles in the human body are TGs and cholesterol, which play crucial roles in various physiological processes (Huang et al., 2022). However, these lipids are insoluble in water and, therefore, must be transported through the bloodstream in the form of lipoprotein complexes (Wiese et al., 2022). Plasma lipoproteins are enveloped by phospholipids, unesterified cholesterol, and apolipoprotein, and their core is composed of TGs and cholesterol (Jaragh-Alhadad et al., 2022). The term ‘lipoprotein’ represents a specific fusion of ‘lipids’ and ‘proteins’, and each complex is distinguished by its lipid composition, density, and surface presence of apolipoprotein. The main categories of lipoproteins include chylomicrons, very low-density lipoprotein (VLDL), intermediate-density lipoprotein (IDL), low-density lipoprotein (LDL), and high-density lipoprotein (HDL). Each type of lipoprotein varies in size, density, and the composition of its lipid core. Chylomicrons and VLDL are the largest and most buoyant particles, characterized by a core that is rich in TGs. In contrast, LDL and HDL contain a higher proportion of cholesterol in their cores and exhibit increased particle density (Deng et al., 2022).

#### Chylomicron, very low-density lipoprotein, and intermediate-density lipoprotein

Chylomicrons are formed in the intestinal cavity during the digestion and absorption of fats. These lipoproteins are the largest type and are particularly rich in TG. Once formed, chylomicrons travel through the bloodstream to various tissues, including skeletal muscle, adipose tissue, and the liver. These tissues contain high concentrations of lipoprotein lipase (LPL) within their capillary beds. LPL plays a crucial role in hydrolyzing the TGs within chylomicrons into free fatty acids. Alternatively, the fatty acids can be metabolized in the liver, kept in adipose tissue, or utilized for the synthesis of VLDL in the liver (Wuni et al., 2022).

VLDL shares similarities with chylomicrons and is characterized by high levels of TG. LPL hydrolyzes the TG component of VLDL, releasing fatty acids for utilization by adipose and muscle tissues. The remaining is termed IDL, which can be converted into LDL through the enzymatic activity of liver lipase or internalized by the liver (Chang et al., 2022).

#### Low-density lipoprotein

LDL particles are primarily responsible for transporting cholesterol throughout the bloodstream and delivering it to various cells (Zhang et al., 2022). These peripheral cells utilize cholesterol from LDL to construct cellular membranes and synthesize hormones (Jiang et al., 2022). As a class of atherogenic lipoprotein particles, elevated levels of LDL are associated with an increased risk of cardiovascular and cerebrovascular diseases (Kim et al., 2022). Furthermore, variations in cholesterol content among LDL particles lead to differences in size, which is a critical factor in the risk of atherosclerosis linked to LDL (Tsai et al., 2015). Although the precise mechanisms remain unclear, smaller, denser LDL particles (phenotype B), which contain a higher cholesterol content, are believed to be more atherogenic compared to larger, less dense LDL particles (phenotype A) (Masip et al., 2022). Additionally, it is suggested that small, dense LDL may be more susceptible to oxidative modifications, making it more detrimental to the vascular endothelial cells. A cascade of immune and inflammatory processes occurring in the arterial wall significantly conduces to the initiation and progression of atherosclerosis, ultimately causing vascular events like myocardial infarction, and cerebral infarction (Moldovan et al., 2022).

#### High-density lipoprotein

HDL are recognized for their ability to reduce the likelihood of cerebral infarction, whereas decreased HDL levels heighten this risk. The protective role of HDL involves facilitating the transport of cholesterol from lipoproteins and peripheral tissues to the liver. HDL is metabolized and synthesized in both the intestine and liver. In newborns, free cholesterol in peripheral tissues is extracted by HDL through the action of two specific enzymes. One of these enzymes is lecithin-cholesterol acyltransferase, a circulating enzyme that enhances the uptake of free cholesterol by HDL through the process of esterification, converting free cholesterol into cholesterol esters. The other enzyme is cholesterol ester transfer protein, which facilitates the exchange of neutral fats and phospholipids among plasma lipoproteins (Formanowicz et al., 2022).

#### Lipoprotein (a)

Lipoprotein (a) (Lp(a)) is a unique lipoprotein particle that shares structural characteristics with LDL. The inclusion of the apolipoprotein A (ApoA) component links Lp(a) to lipid metabolism and the blood coagulation process. Structurally, Lp(a) resembles both plasminogen and LDL, and it is believed that this particle has the potential for both atherogenic and thrombogenic activity (Mohammed et al., 2022).

### Apolipoproteins

Apolipoproteins, such as ApoA, apolipoprotein B (ApoB), apolipoprotein C (ApoC), and apolipoprotein E (ApoE), encapsulate lipoprotein particles responsible for lipid transport within the bloodstream and facilitate the recognition of these particles by enzymes that metabolize or eliminate lipids (Tan and Ong, 2025). For example, ApoC-2 enhances the activity of LPL, which assists in the removal of TGs from lipoprotein particles, including chylomicrons and VLDL (Li et al., 2022b). ApoC-3 is an LPL antagonist (K et al., 2020).

Apolipoproteins play various roles in lipid metabolism. ApoE (E2, E3, and E4) facilitates the uptake of remnant particles, including chylomicron remnants, VLDL, and IDL remnants. ApoB-100 and ApoB-48 are present in VLDL and LDL and act as ligands for the LDL receptor (LDLR) in chylomicrons and intestinal cells (Botha et al., 2022). ApoA-2 is found in both chylomicrons and HDL particles, stimulating the lecithin cholesterol acyltransferase enzyme and contributing to the structural integrity of HDL particles (Puig et al., 2025).

### Lipid metabolism enzymes

Several key enzymes have been identified as crucial players in lipoprotein metabolism, activated by apolipoproteins to execute specific functions within this biological process. One prominent enzyme, LPL, is responsible for hydrolyzing TG in chylomicrons and VLDL. This enzymatic action is vital for the breakdown of fats, allowing their utilization by the body. Another important enzyme in this metabolic pathway is lecithin-cholesterol acyltransferase, which esterifies free cholesterol present on the surface of HDL (Miura et al., 2022). By facilitating this process, lecithin-cholesterol acyltransferase plays a key role in maintaining cholesterol homeostasis and promoting the reverse transport of cholesterol (Norum, 2017). Additionally, hepatic TG lipase hydrolyzes TGs within IDL and HDL particles, further contributing to lipid metabolism and the regulation of lipid levels in the bloodstream (Nakajima et al., 2018). Through these enzymatic activities, the body efficiently manages lipoprotein metabolism, which is essential for overall health.

### Lipid transport pathway

Three primary methods for the production and transportation of lipids exist the exogenous pathway, the endogenous pathway, and the cholesterol reverse transport pathway (Chung et al., 2022).

#### Exogenous (dietary) pathway

After digesting and absorbing dietary fat, TG and cholesterol are converted into chylomicrons in intestinal epithelial cells. The chylomicrons enter the intestinal lymphatic system, initiating their journey through the body. Once in the bloodstream, circulating chylomicrons interplay with capillaries in muscle cells and adipose tissue. During this interaction, TG is released into adipose tissue, serving both as a storage form and as a source of energy to meet the metabolic demands of the body. Meanwhile, the residual particles remaining from chylomicrons after their lipid content has been utilized are efficiently cleared from the bloodstream. This clearance is accomplished through specific receptors located in the liver that recognize and bind to chylomicron remnants, facilitating their removal from circulation (Huh et al., 2022).

#### Endogenous pathway

Endogenous pathway depends on the liver’s synthesis of lipoproteins. The liver produces TG and cholesterol esters, which are assembled into VLDL particles and subsequently released into the bloodstream. These VLDL particles are hydrolyzed by LPL into fatty acids and glycerol. Muscle cells utilize the fatty acids for energy, while adipocytes store them. Following LPL processing, a VLDL particle transforms into a VLDL remnant. Most of these residues are absorbed by the liver through LDLRs, while the remaining remnants are converted into IDL. A portion of IDL is reabsorbed by the liver (also through LDLRs), while other IDLs are hydrolyzed by hepatic TG lipase in the liver, leading to the formation of LDL. Cholesterol in the body is primarily transported by LDL, which is utilized by extrahepatic cells for the synthesis of steroid hormones and intercellular membranes. Most LDL particles are absorbed by receptors in the liver, while the remainder is cleared via the scavenger pathway (Meng et al., 2022). When a cell absorbs LDL, free cholesterol is discharged and begins to accumulate within the cell.

The concentration of plasma LDL is regulated through the modulation of LDLR activity and the uptake of LDL. This regulation entails the production of hydroxy-3-methylglutaryl-coenzyme A reductase, which controls the cholesterol synthesis rate. Additionally, it involves the suppression of new LDLR formation in cells and the activation of the enzyme acyl-coenzyme A cholesterol acyltransferase, which facilitates the conversion of free cholesterol into cholesterol esters. Acyl-coenzyme A cholesterol acyltransferase plays a crucial role in the intracellular storage of cholesterol (Bhattacharjee et al., 2022).

#### Cholesterol reverse transport pathway

The clearance of cholesterol from tissues and its transport back to the liver is called cholesterol retrograde transport. A key lipoprotein involved in this process is HDL, which also facilitates the conversion of cholesterol esters among various lipoproteins. HDL particles have multiple subtypes including HDL2 and HDL3 (Hintikka et al., 2022). Hepatic and intestinal tissues are primarily responsible for the synthesis of HDL. The newly formed HDL by the liver is predominantly composed of phospholipids and ApoA-1. Through the action of lecithin cholesterol acyltransferase, free cholesterol is converted into cholesterol esters, facilitating the maturation of lipoproteins into HDL3, which subsequently transforms into HDL2 with the involvement of LPL. HDL plays a crucial role in transporting free cholesterol from peripheral tissues to various cells, predominantly the liver, by interacting with circulating lipoproteins or specific macromolecules, thereby enabling reverse cholesterol transport (Ouimet et al., 2019). Reverse cholesterol transport assists in the clearance of cholesterol from tissue cells and helps maintain a relative balance of cholesterol levels within cells, thereby mitigating the risk of atherosclerosis development (Xu et al., 2022). This mechanism offers a protective effect against atherosclerosis. Consequently, elevated levels of HDL are considered to confer a protective effect. It is now recognized that lipoproteins other than HDL, and residual particles resulting from lipid metabolism, possess a higher atherogenic potential. To address this issue, the term “non-HDL cholesterol” has been introduced to encapsulate the heightened risk present in lipid profiles, which may not be fully recognized through LDL assessment alone. Consequently, non-HDL cholesterol serves as a more comprehensive marker for evaluating cardiovascular disease risk. This marker is crucial for ensuring that patients receive appropriate treatment to achieve target cholesterol levels (Raja et al., 2023).

## Lipid Metabolism, Atherosclerosis, and Ischemic Stroke

The brain has a notably high concentration of lipids, particularly polyunsaturated fatty acids (PUFAs), which serve as vital bioactive nutrients for brain development, memory capacity, and learning (Hamed et al., 2022). Lipids are also pivotal for maintaining the structural integrity of brain cells, influencing membrane fluidity and permeability, facilitating energy metabolism, and supporting signaling pathways (Hoscheidt et al., 2022). Lipid metabolism disorders have increasingly contributed to the development and pathogenesis of stroke in recent years. Hyperlipidemia is one of the key risk factors for stroke (Guzik and Bushnell, 2017). Elevated intracellular calcium levels, coupled with oxidative stress resulting from ischemia-reperfusion in rats, activate enzymes such as calcium-dependent cytoplasmic phospholipase A2, leading to a rapid release of arachidonic acid (Rieg et al., 2018). Hypoxic conditions within brain tissue exacerbate the release of PUFAs from phospholipids. Furthermore, this release has been shown to be significantly increased in a mouse model following ischemic stroke (Morillo-García et al., 2014).

### Atherosclerosis and lipid metabolism

Atherosclerosis, a condition characterized by the accumulation of plaque within the arteries, often directly evolves to ischemic stroke. The formation of atherosclerotic plaque involves three critical processes: the initial development of fatty streaks, the progression of atherosclerosis, and the rupture of atherosclerotic plaques (Wei et al., 2022; Ding et al., 2024).

The development of fat streaks can be categorized into three distinct processes: the capture of LDL, the activation of endothelial cells and leukocytes, and the formation of foam cells. Endothelial dysfunction, which result from hypertension and elevated cholesterol levels, leads to increased permeability of LDL to the tunica intima (Khatana et al., 2020). This heightened permeability facilitates the retention of extracellular proteoglycans within the extracellular matrix. The accumulation of these proteoglycans further enhances the retention of LDL at the site of the lesion, where retained LDL may undergo oxidation upon exposure to reactive oxygen species (ROS), ultimately resulting in the formation of oxidized LDL (Khatana et al., 2020).

Oxidative LDL has been shown to promote monocyte binding to endothelial cells in a mechanism independent of chemokines and adhesion molecules. To increase atherosclerotic burden, oxidative LDL not only binds to scavenger receptors, but also is able to bind to other classes of pattern recognition receptors of immune cells (Rhoads and Major, 2018). Monocytes differentiate into macrophages, which, along with smooth muscle cells, engulf oxidized LDL, contributing to foam cell formation. When these foam cells undergo apoptosis, the lipids they contain become trapped within the intima, thereby triggering an inflammatory response (Wolf and Ley, 2019).

In response to inflammation, endothelial cells release adhesion molecules and chemotactic agents that further recruit monocytes and T lymphocytes. Conditions that stimulate the proliferation of smooth muscle cells also facilitate their migration toward the vessel lumen, where they proliferate alongside macrophages and T lymphocytes. The increase in collagen fiber density can activate the extracellular matrix, thereby facilitating the development of atherosclerotic plaques. Tumor necrosis factor (TNF) alpha and protease released by T lymphocytes and macrophages, respectively, can compromise the structural integrity of atherosclerotic plaques, making them susceptible to rupture. However, the atherosclerotic plaque, shielded by a substantial fibrous cap, continues to enlarge until it obstructs blood flow, ultimately leading to the outcome of ischemic. Additionally, fatty acids derived from TGs contribute to the progression of atherosclerosis (Odukoya et al., 2021).

### Ischemic stroke-related lipid metabolism gene

In ischemic stroke, we focus on lipid metabolism genes such as proprotein convertase subtilisin/kexin type 9 (PCSK9), APOB, APOE, lipoprotein (A) (LPA), cholesteryl ester transfer protein (CETP), and ATP binding cassette subfamily A member 1 (ABCA1). These genes are associated with ischemic stroke and play a critical role in the formation of atherosclerotic plaques.

#### PCSK9 gene

The critical role of PCSK9 in regulating LDL metabolism was identified through a gain-of-function mutation found in a French family with familial hypercholesterolemia. These mutations, particularly missense single nucleotide polymorphisms, are believed to elevate both the expression levels and enzymatic activity of the PCSK9 protein. Consequently, there is an increased degradation of LDLRs, resulting in a diminished presence of LDLR on the membranes of liver cells, which in turn reduces the efficacy of LDL clearance. The accumulation of lipid metabolites in circulation can lead to hypercholesterolemia, which contributes to the formation of atherosclerotic plaques and may heighten the risk of ischemic stroke (Johannesen et al., 2022). Furthermore, studies have indicated that loss-of-function mutations in PCSK9 can enhance LDLR function and decrease LDL cholesterol levels (Mikaeeli et al., 2020).

#### APOB gene

The *APOB* gene encodes ApoB protein, which is located in the p24–p23 region of chromosome 2. ApoB is a glycoprotein that plays an important role in the secretion and assembly of chylomicrons and VLDL from the liver and small intestine. Furthermore, ApoB is vital for maintaining the structural integrity of VLDL and LDL particles (Whitfield et al., 2004). A study involving 595 Slovenians revealed that individuals with the *APOB* rs693 TT genotype exhibited a 1.74-fold increased risk of developing carotid plaque. In contrast, those carrying the *APOB* rs1042031 AA genotype showed a significantly lower likelihood of carotid plaque formation (Boekholdt et al., 2003). Additionally, a missense mutation in *APOB* rs1042034, resulting in the Ser4338Asn substitution, along with the T allele variant and the G allele variant of *APOB* rs673548, may heighten the risk of ischemic stroke in the Chinese Han population (Zhou et al., 2018).

Conversely, the presence of *APOB* SpIns/Del mutations may affect the structure of signaling peptides, thereby influencing lipid metabolism. A study conducted on European Atherosclerosis, which involved 682 patients with coronary heart disease and 1312 healthy controls, revealed that plasma cholesterol concentrations tend to increase among carriers of the SpIns/Del polymorphic D allele. Furthermore, individuals possessing the SpIns/Del polymorphic DD genotype exhibited significantly higher levels of LDL-C and ApoB (Boekholdt et al., 2003).

#### APOE gene

*APOE* is recognized as one of the most extensively studied candidate genes associated with stroke. It plays a critical role in lipid transport and metabolism (Lai et al., 2020). This gene, located on the long arm of chromosome 19 (19q13.2), is clustered with several APOC genes. The human *APOE* gene is polymorphic, comprising three common alleles (ε2, ε3, and ε4) that encode distinct subtypes of APOE (Wang et al., 2019a). The ε4 allele has been associated with elevated total cholesterol levels, whereas the ε2 allele is correlated with reduced cholesterol levels. A study on a large population cohort of French elderly showed that individuals carrying the ε34 genotype exhibit increased intima-media thickness of common carotid arteries, but the association was only marginal (Debette et al., 2006).

Research examining the relationship between genetic polymorphisms and stroke has yielded inconsistent findings. While a significant association between *APOE* gene polymorphism and ischemic stroke was not detected in the European population (Casas et al., 2004), a moderate correlation was identified in Asian populations (Banerjee et al., 2007). Both the ε2ε3 genotype and the ε4 allele exhibit increased expression in individuals suffering from ischemic stroke, as indicated by smaller case-control and cross-sectional studies (Couderc et al., 1993; de Andrade et al., 1995; Schmidt et al., 1997). Furthermore, other researchers have investigated the influence of the *APOE* genotype on the outcomes of cerebral infarction. Given the crucial role this lipoprotein plays in lipid metabolism within the brain, McCarron et al. (1999) discovered that the ε4 allele positively impacts stroke prognosis. Meanwhile, Qiao et al. (2022) analyzed the contradictory results concerning the significance of *APOE* alleles in susceptibility to ischemic stroke.

#### LPA gene

Growing evidence indicates that high concentrations of Lp(a) play a significant role in the development of stroke (Arora et al., 2019). Genetic studies have demonstrated that LPA is a hereditary trait primarily determined by the APOA locus. Variations within the *APOA* locus, particularly those outside the kringle IV-2 domain, appear to influence Lp(a) levels (Lampsas et al., 2023). Notably, variants of the pentanucleotide TTTTA repeat located in the 5′ untranslated region of the *APOA* gene account for 10% to 14% of the variability observed in plasma Lp(a) levels and exhibit an inverse correlation with Lp(a) concentrations (Ogorelkova et al., 2001). In three independent studies, a reduced number of *APOA* TTTTA variable number tandem repeats was associated with ischemic stroke (Hu et al., 2000; Liu et al., 2002; Sun et al., 2003).

#### CETP gene

This glycoprotein, primarily synthesized by the liver and present in plasma, primarily interacts with HDL, facilitating the transfer of cholesterol esters from anti-atherosclerotic HDL to pro-atherosclerotic apolipoproteins. *CETP* gene is located on chromosome 16q21 and consists of 16 exons. Numerous polymorphisms have been identified, including Taq 1b in intron 1, 405 V and A373 P in exon 12, as well as R451Q and -629A/C in exon 15 (Morton et al., 2019).

#### ABCA1 gene

The ABCA1 protein plays a crucial role in the reverse transport of cholesterol. As a membrane transporter, it facilitates the efflux of cholesterol and phospholipids toward ApoA. Furthermore, it functions as an enzyme involved in the turnover of cholesterol and phospholipids at the plasma membrane. The proper functioning of ABCA1 is essential for preventing cholesterol accumulation in macrophages, which can occur due to the oxidation or modification of LDL. In fact, the efficiency of lipid efflux is diminished due to impaired interactions between ABCA1 and ApoAI, a condition that arises from mutations in the first and second extracellular loops of the ABCA1 protein. Consequently, the abnormal activity of ABCA1 may result in ineffective cholesterol efflux and could contribute to the development of precursors to arterial disease, such as foam cells (Singaraja et al., 2003). In Tangier disease, the deficiency of ABCA1 is prevalent, resulting in a diminished capacity of cells to effectively transport cholesterol and phospholipids, which leads to an accumulation of cholesterol in peripheral tissues (Fitzgerald et al., 2010). Consequently, research suggests that variations in the *ABCA1* gene may contribute to the observed differences among individuals in terms of their susceptibility to and severity of atherosclerosis.

## Stroke-induced Immune Responses

### Ischemic stroke

Neuroinflammation and repair are important processes following both ischemic stroke and intracerebral hemorrhage. In both diseases, neuroinflammation is featured by firstly activating innate immune, including microglia and astrocytes. In ischemic stroke, inflammation is initiated by a lack of blood supply, while in hemorrhage, inflammation is induced by leaked blood products. Ischemic stroke evokes innate immunity as well as adaptive immunity, which plays key roles both in the acute and chronic phases of cerebral ischemia (Pandolfi et al., 2025). Once ischemia occurs, a complicated innate immune response is triggered. The pro-inflammatory signals activate microglia and astrocytes rapidly, induce cytokines and chemokines production and cause infiltration of a wide range of immune cells into the ischemic lesion (Dugue et al., 2017). After acute cerebral ischemia insult, a batch of hazardous events occurs immediately, leading to death of neurons and releasing danger-associated molecular patterns molecules (DAMPs) (Chamorro et al., 2016; Endres et al., 2022). This pathological process causes more neuronal cell death and even exacerbates the cerebral ischemic damage.

Microglia start to respond minutes after stroke in both the ischemic core and the penumbra region. In the acute phase, microglia in the core lesion detect DAMPs by their receptors which mainly belong to the Toll-like receptors (TLRs) family (Liesz et al., 2015; Gülke et al., 2018). Once activated, microglia facilitate the expression of CD68, CD45 and CD11b that relate to a phagocytic phenotype contribute to cell debris clearance. They also express TNF and the pro-inflammatory interleukins (ILs) that activate astrocytes and endothelial cells (Xu et al., 2020). Afterwards, DAMPs and cytokines enter the peripheral circulation through the disrupted blood–brain barrier (BBB) and the cerebrospinal fluid drainage system (Anrather and Iadecola, 2016). They induce peripheral immune cells to infiltrate brain lesion and lead to the ensuing inflammatory responses, which can further facilitate releasing of proteolytic enzymes and ROS that break the BBB, leading to serious endothelial injury and hemorrhagic transformation (Perez-de-Puig et al., 2015; Zhang et al., 2021a). Microglia with a proliferation profile generate insulin growth factor-1 (IGF-1) related to a pro-repair function that continue 1–2 weeks after stroke onset, while pro-inflammatory microglial have been reported to be persistent for weeks post-ischemia (Planas, 2024). The innate immunity runs throughout the acute phase, subacute phase and even the chronic recovery phase.

In contrast with innate immunity, adaptive immunity needs several days to evolve and reserves immunological memory of pathogens exposure in chronic phase (Jain and Pasare, 2017). Adaptive immunity is based on immunoglobulins and T cells. According to mouse and human autopsy samples, the brain continued to amass T cells 1 month post-stroke (Heindl et al., 2021). T helper (Th) 2 and regulatory T cells release cytokines like IL-4, IL-5, IL-10, and IL-13 to facilitate brain repair, and also contribute to inflammation resolution (Ito et al., 2019). CD8^+^ T cells, Th1, Th17, and γδT cells may aggravate ischemic damage by releasing cytokines such as interferon (IFN)-γ, IL-2, IL-12 and IL-17 30 days post-stroke (Xie et al., 2019).

The precise mechanism of the dual properties of inflammation in cerebral ischemia has not been fully understood yet. However, in some studies, blocking certain points of the inflammatory pathway in innate immunity or adaptive immunity has been shown to reduce infarct size in stroke models (Liu et al., 2020; Cao et al., 2023), while in others, inhibition of inflammatory targets, such as IL-6 and TNF-α, has been shown to exacerbate disability in experimental stroke models (Liguz-Lecznar and Kossut, 2013; Jayaraj et al., 2019).

### Cerebral hemorrhage

When intracerebral hemorrhage strikes, the process of the ongoing inflammatory response is similar to ischemic. Microglia transformation is trigged, secreting proinflammatory cytokines like TNF-α and IL-1β, breaking up the BBB and inducing neuronal apoptosis. Microglia activation presents with increasing phagocytic receptors, inflammatory cytokines and their morphology change into an ameboid style (Xue and Yong, 2020). Levels of inflammatory factors and the proportion of pro-inflammatory microglia are increased, and neuronal apoptosis is induced in mice at 24 hours following subarachnoid hemorrhage (Gao et al., 2021). Matrix metalloproteinases (MMPs) and the transcription factor nuclear factor κB (NF-κB), are both markers of microglia orient to adverse results following intracerebral hemorrhage (Zhang et al., 2015; Lattanzi et al., 2020). Within 3–7 days of attack, the microglia are found to initiate the conversion of protective phenotype, contributing to hematoma clearance, tissue regeneration, and overall inflammation resolution. The repair process of hemorrhage is supported by the release of more anti-inflammatory cytokines by the Th2 and regulatory T cells (Tschoe et al., 2020). Lower pro-inflammatory microglia level and higher IL-­10 concentration are related to better neurological function in 90 days (Zhang et al., 2015). The elevated transforming growth factor β1 (TGF­β1) level is also associated with a better prognosis. These beneficial factors of neuroinflammation that are remarkedly increased in recovery periods of cerebral hemorrhage are accompanied by progressively increased regulatory T cells (Taylor et al., 2017).

In contrast to ischemia, extreme cerebral edema and hematoma which is unique in intracerebral hemorrhage are also associated with neuroinflammation. The microglia involved inﬂammation processes of hemorrhage act as a double‐edged sword. The rapid accumulated blood-derived products (e.g., hemoglobin, heme, and iron) activate microglia and potent inflammatory response that induce worsening cerebral edema surrounding hematoma (Wang et al., 2019b). Microglia-related sustained inﬂammation results in neurologic deterioration (Vinukonda et al., 2019). Simultaneously, as early as up to 1 hour after attack, microglia which shift to a protective phenotype were seen to demonstrate repair action to hemorrhagic damage (Chang et al., 2017). The hematoma clearance ability of microglia might provide pivotal support for recovery from hemorrhagic damage. Activated microglia also can phagocytose the hematoma, reduce brain swelling and mitigate neurological deﬁcits (Jing et al., 2019). Otherwise, hematoma absorption may also produce a series of immune reactions leading to brain injury (Zhang et al., 2017). Microglia-associated autophagy would contribute to inﬂammation and brain damage as well as repair in intracerebral hemorrhage (Xiao et al., 2020). TLR4-mediated autophagy of microglial activation leads to inﬂammatory actions causing secondary brain damage and repair (Liu et al., 2021). The mechanism of autophagy is mazy and has not been definite whether it plays a beneﬁcial or harmful role in intracerebral hemorrhage.

The sustained process of inflammation after cerebral hemorrhage provides an opportunity for therapies. Milk fat globule epidermal growth factor-8 lets the microglia shift to protective phenotype, decreasing edema area and reducing neurological dysfunction remarkably after subarachnoid hemorrhage (Gao et al., 2021). Microglia-produced IL-1β has been proven to contributory to cerebral hemorrhagic injury. IL-1β and IL-18 could be produced by the activation of pyrin domain containing 3 (NLRP3) inflammasome, leading to increase of pro-inflammatory cytokines. IL-1Ra is a naturally occurring protein that curbs IL-1β activity via binding to its receptor. Therefore, IL-1Ra would be a potential therapeutic tactic in intracerebral hemorrhage due to its important role in inflammation (Alsbrook et al., 2023). IL-10 treatment significantly relieves functional impairments and promotes the resolution of hematoma in the acute phase of cerebral hemorrhage by curbing microglia activation and inhibiting pro-inflammatory factors release (Han et al., 2023).

### Stroke-related microglia phenotype and function

Microglial cells, the main resident innate immune cells in the brain are pronounced activated early after stroke, ahead of the infiltration of other blood-derived immune cells (Hu et al., 2012; Tschoe et al., 2020; Pluta et al., 2021). The activation of microglia can be a double-edged sword in brain (Shichita et al., 2023). They may participate in all stages of ischemic stroke by releasing pro-inflammatory mediators, including IL-1β, proteolytic enzymes (MMP-9, and MMP-3), TNF-α, IL-6, IFN-γ, and inducible nitric oxide synthase (NOS) (Pluta et al., 2021). The activated inflammation and immune reactions can exacerbate brain damage after stroke (Chu et al., 2015; Maida et al., 2020; Sanchez-Bezanilla et al., 2021). By contrast, microglia may also engulf cell debris and release anti-inflammatory cytokines and growth factors to promote the repair and regeneration of brain tissue after cerebral ischemia (Lu et al., 2005; Singhal and Baune, 2017). Microglial secrete trophic factors involving TGF-β, IGF-1, and vascular endothelial growth factor. Among them, IGF-1 is a key growth factor because it has a beneficial role in neural plasticity, neurite outgrowth and synaptogenesis for cerebral structure remodeling (Ma et al., 2017; Kohno et al., 2022).

Microglia were used to be categorized into “M1” and “M2” based on the expression of markers and indirectly assumed a detrimental (“M1”) or beneficial (“M2”) role (Lan et al., 2017a). However, it has become noticeable that microglia are even more complicated than rigid “M1” and “M2” (Martinez and Gordon, 2014). Microglia are extremely dynamic and constantly responding to episodes of health or disease by adapting diverse states and fulfilling different functions (Vay et al., 2018; Tremblay et al., 2020). Stroke also induces many kinds of stimuli, developing with time and exerting different microglia phenotypes of those showing pro-inflammatory and dysfunctional features or others exhibiting pro-regeneration traits (Planas, 2024). M1 polarization occurs early upon activation in the acute phase of both ischemic stroke and intracerebral hemorrhage, but the evolvement of microglia is different between them. Microglia expressed M1 markers such as CD16, CD32 and inducible nitric oxide synthase prominently from day 3 to day 14 following ischemic stroke. M2 markers such as CD206, arginase-1, Ym-1/2, IL-10, TGF-β is expressed on day 1, peaks within days 3 to 5, and returns to baseline since day 14 (Hu et al., 2012). Correspondingly, in hemorrhage models, M1 markers are rapidly increased within 6 hours, highly expressed from 1–3 days and slowly decreased over 14 days (Lan et al., 2017b), whereas markers of M2 are expressed with low levels at 1 day and increasingly expressed over 14 days after attack (Lan et al., 2017a; Zhang et al., 2017). A small group of M1 cells shift to a primarily phagocytic phenotype (M2) transiently, consuming necrotic cells and cellular debris to reduce the release of inflammatory cytokines in acute phase (Gomez-Nicola and Perry, 2015). However, the M1 microglia are increasing with time, the phagocytic ability of microglia decreases significantly, and the levels of inflammatory cytokines and chemokines increase instead, causing broad tissue damage. IL-4 is the assumed cytokine that alters microglia state from the pro-inflammatory to the tissue restorative phenotype and improves functional outcomes after intracerebral hemorrhage (Liu et al., 2016, 2024a). TGF-β and IL-10 also improve the protective function of microglia (Bedolla et al., 2024; Bido et al., 2024).

The supposed classification of M1 and M2 microglia is considered to be oversimplified now. These two extreme activation phenotypes can merely be found *in vitro*, while the situation *in vivo* is more complicated. Integrative analyses like single-cell technologies, multi-omics, are applied to gene, protein and metabolism levels which helps to draw a profile of rich diversity of microglia subtypes. A large amount of diverse microglial phenotypes in a variety of given background have been perceived in health brain and disease models. The disease-associated microglia (DAM) originated from Alzheimer’s disease (AD) models but was then recognized to be related to ischemic stroke and cerebral hemorrhage (Keren-Shaul et al., 2017; Wlodarczyk et al., 2017; Cao et al., 2021; Paolicelli et al., 2022; Zhu et al., 2024). Because it was found that gene expression in microglia of ischemic brain was parallelly upregulated or downregulated with the DAM. Besides, microglia acquired traits like DAM within hours or days after an acute ischemic stroke insult (Arbaizar-Rovirosa et al., 2023). A unique group of stroke-associated myeloid cells identified by transcriptional analysis also shared some similarities with certain subsets of DAM (Keren-Shaul et al., 2017). Stroke-associated myeloid cells were found in different stroke models across different species, up-regulating genes of lipid metabolism and expressing a phenotype of phagocytosis (Beuker et al., 2022). It has been also figured out that some DAM-expressed genes participate in lipid transport and metabolism (Keren-Shaul et al., 2017; Krasemann et al., 2017; Deczkowska et al., 2018). Furthermore, the microglia most closely related to lipid metabolism are lipid-droplet accumulating microglia (LDAM), which are associated with stroke and aging (Marschallinger et al., 2020). LDAM induce impairing of phagosome maturation, increasing of oxidant production and excessive release of pro-inflammatory cytokines (Chausse et al., 2021). LDAM increased 8.3-fold in stroke model mice while the proportion of LDAM in old stroke model mice further increased by 2.7-fold (Marschallinger et al., 2020).

Recently, a sub-cohort of CD11c^+^ microglia was discovered for their contribution to the repair of stroke injury by single-cell transcriptomics (Jia et al., 2023). In neurodegenerative disease, DAM which is involved in lipid metabolism and phagocytosis was CD11c positive. They were presumed to be able to take part in restricting neurodegeneration although the mechanism has not been found (Keren-Shaul et al., 2017; Deczkowska et al., 2018; Cao et al., 2021; Zheng et al., 2021). Only a small portion of CD11c^+^ cells are resident in the healthy brains of adult mice. In case of brain hurt, CD11c^+^ cells demonstrate a pro-repair and myelin-supportive gene feature, proliferating and participating in remyelination and immune activation (Keren-Shaul et al., 2017; Wlodarczyk et al., 2017; Cao et al., 2021). CD11c^+^ microglia keep expanding till day 30 after middle cerebral artery occlusion (MCAO), which is consistent with ischemic white matter recovery period (Jia et al., 2023). CD11c^+^ cells in the injured tissue demonstrated high phagocytotic ability and expressed lipid metabolism-associated genes may engulf myelin and promote remyelination after ischemic stroke (Gallizioli et al., 2020). Depletion of CD11c^+^ cells in the recovery phase of stroke can prevent myelin recovery and worsen functional outcomes (Jia et al., 2023). Phagocytosing too much lipid-rich myelin debris may lead to cholesterol overload. Excess cholesterol flows out through translocators such as ApoE and ApoC-1 to keep cholesterol homeostasis (Hong and Tontonoz, 2014; Dai et al., 2021). Myelin-clearance by CD11c^+^ cells might cause a compensatory increase in the production of apolipoprotein, which could help cholesterol recycling and remyelination (Natrajan et al., 2015; Berghoff et al., 2021).

## Microglia and Lipid Droplets in Stroke

**Figure 1:**
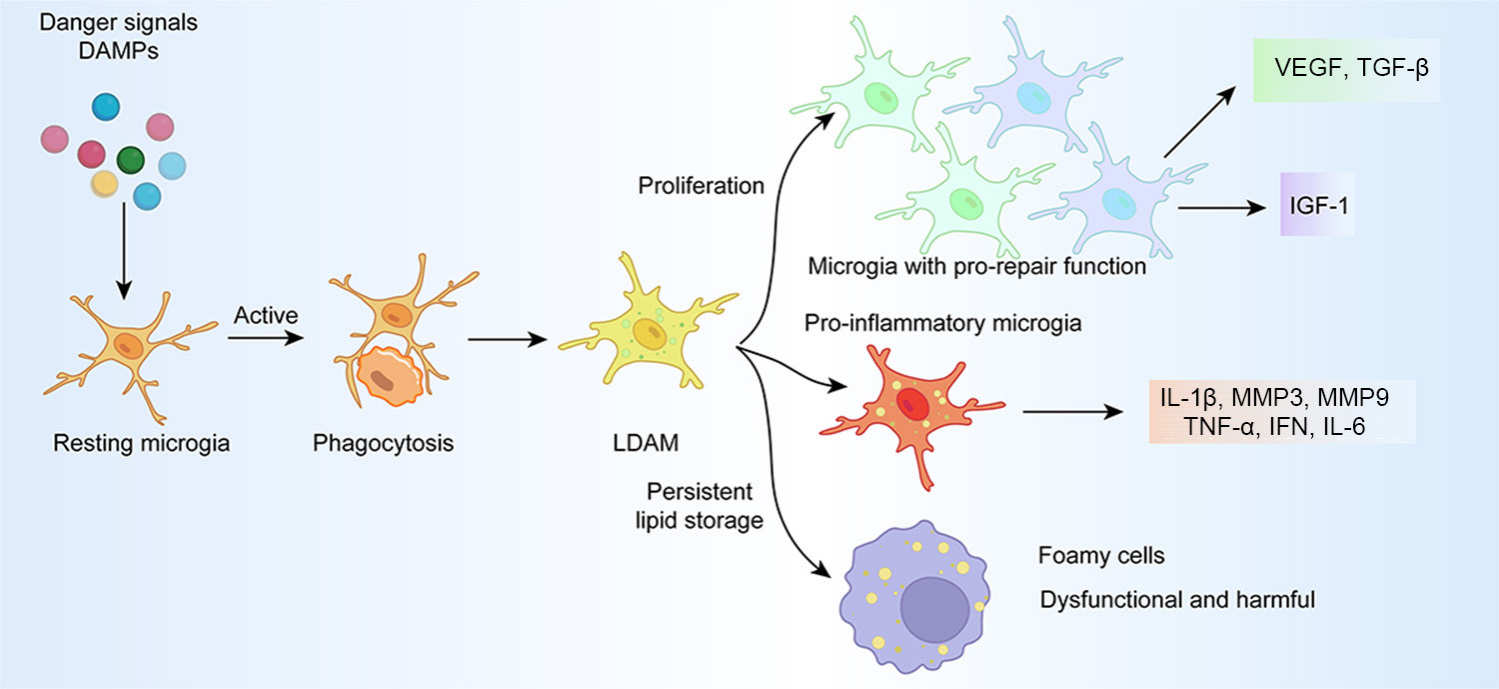
LDs involved microglia reactions to experimental brain ischemia. Microglia are activated after ischemia. The injured tissue promotes microglia phagocytosis and accumulates LDs in them. Then microglial generate diverse phenotypes and functions. Some cells undergo inflammasome activation and IL-1β, TNF-α, and IL-6 production that triggers further inflammatory profiles, while others display a pro-repair function. Fat storage can support cell proliferation. Proliferating microglia produce IGF-1 associated with pro-repair functions. The microglial cells that continue to amass lipids, instead of utilizing them, show larger LDs and generate dysfunctional foam cells. IFN: Interferon; IGF-1: insulin growth factor-1; IL: interleukin; LD: lipid droplet; LDAM: lipid-droplet accumulating microglia; MMP: matrix metalloproteinase; TNF-α: tumor necrosis factor α; VEGF: vascular endothelial growth factor.

Recently, research has suggested a putative role for lipid metabolism in the regulation of microglia function, including microglial activation, proliferation, phagocytosis, and inflammation signal transduction (Krasemann et al., 2017; Chausse et al., 2021). Stroke-induced activation in microglia is highlighted with lipid metabolism involving lipid droplet (LD) aggregation (Lin et al., 2019; Arbaizar-Rovirosa et al., 2023). LDs are small organelles that store fatty acids and have a variety of regulatory functions (Bosch et al., 2020; Planas, 2024). On the 3^rd^ day of ischemia, microglia gather at the periphery of focal ischemic lesion and inflammation arouses a metabolic change favoring lipid synthesis (Arbaizar-Rovirosa et al., 2023). A few studies demonstrated that lipid synthesis metabolism and phagocytic lipid intake of microglia drive LD biogenesis, increase inflammation, and enable microglia proliferation (Vander Heiden et al., 2009; O’Neill et al., 2016). Proliferating microglia secrete trophic factors that contribute to the protection and repair of ischemic tissue. However, some microglia which persistently accumulate LDs transform into dysfunctional and potentially harmful foamy cells (Beccari et al., 2023). LD-rich foamy cells were found 7 weeks after ischemic stroke while therapies reducing lipid accumulation mitigate the prolonged inflammatory response and prompt behavioral improvement (Becktel et al., 2022). In the study of the intervention of LD inhibitor or peroxisome proliferator-activated receptor (PPAR) antagonist, LD formation was prevented, not only the level of inflammation and the death rate of microglia treated with glucose-oxygen deprivation but also the volume of cerebral infarction and motor dysfunction of ischemia models were significantly reduced (Lin et al., 2019; **Figure 1**).

### Microglia sensing and lipid-sensitive receptor

**Figure 2:**
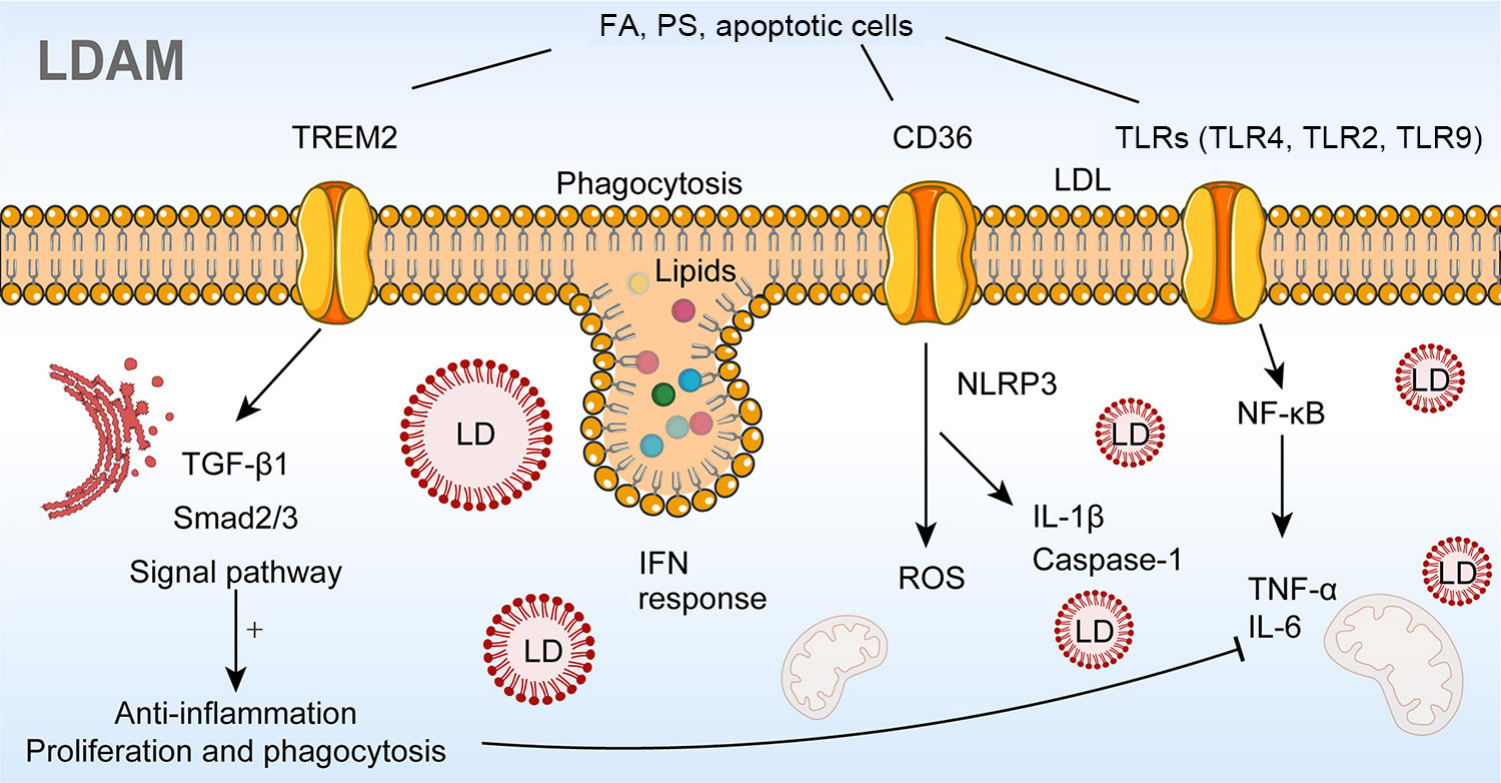
Microglia sensing and lipid-sensitive receptors. Phagocytosis of lipid-rich materials, for example, cell membranes and myelin, generates LDs. Microglia are activated via lipid-sensitive receptor such as TREM2, CD36, TLRs after stroke. The progressive utilization of lipids from LDs triggers several signal cascades ultimately inducing inflammation, anti-inflammation and proliferation functions of microglia. FA: Fatty acid; IFN: interferon; IL: interleukin; LD: lipid droplet; LDAM: lipid-droplet accumulating microglia; PS: phosphatidylserine; ROS: reactive oxygen species; TGF-β1: transforming growth factor β1; TLRs: Toll-like receptors; TNF-α: tumor necrosis factor α; TREM2: triggering receptor expressed on myeloid cells 2.

Microglia are featured of their universal sensitivity to alterations in their local environment, partly via the wide range of receptors expressed at their membrane, known as “microglia sensome.” The complex “sensome” gives microglia the ability to sense lots of signals that allow them to detect changes in their environment (Hickman et al., 2013). Sensing different molecular signals by the microglial sensome, microglia demonstrate a variety of phenotypes to either worsen ischemic damage or oppositely cause repair and regeneration (Patel et al., 2013). Among all receptors, microglia expressed, several of them are lipid-sensitive, such as triggering receptor expressed on myeloid cells 2 (TREM2), CD36, TLRs, receptors for fatty acids derivatives like endocannabinoids, oxylipins, etc. (Mauerer et al., 2009). These receptors can bind to lipid cell membrane components such as fatty acids, phosphatidylserine or oxidized lipids and phagocytose myelin, spines, apoptotic cells, protein aggregates and so on (Xue et al., 2019). Via these receptors, LDs accumulating in microglia shortly after stroke occurrence is plausibly facilitate to the phagocytosis of damaged tissue and immunometabolism disturbances (Arbaizar-Rovirosa et al., 2023; **Figure 2**).

An important lipid-sensitive receptor is CD36, a type B scavenger receptor that identifies LDL, oxidized phospholipids, and amyloid-β. It is also known as a fatty acid transporter or uptake promoter (Kim et al., 2008; Gao et al., 2010). CD36 activation arranges transcriptional and metabolic reshaping in microglia that are involved in the regulation of phagocytosis, activation of innate immunoreaction and production of oxidants (Ioghen et al., 2021; Feng et al., 2024). It was reported that treating with a CD36 inhibitor before acute stroke insult could decrease the infarct size and swelling volume (Kim et al., 2020b). Also, in CD36-null mice the infarct area was reduced and the neurological outcome was promoted. CD36 starts the inflammatory cascade by sensing danger signals triggered by cerebral ischemia (Garcia-Bonilla et al., 2015). The CD36 highly expressed microglia locate in the cerebral ischemic lesion and induce the formation of ROS in the early phase of stroke while CD36 deficiency can reverse the ischemia-increased ROS, reduce the infarct size, and improve the neurological function (Cho et al., 2005). The inflammatory process triggered by microglial CD36 in brain ischemia is critically associated with the regulation of caspase-1 activity and the production of IL-1β (Garcia-Bonilla et al., 2021). The production of active IL-1β attributes to priming IL1b transcription and inducing assembly of the NLRP3 inflammasome complex by CD36 (Kagan and Horng, 2013). Combined with oxidized LDL, CD36 also stimulates inflammation by induction of TLRs assembling and the internalizing of receptor-ligand complexes (Stewart et al., 2010; Sheedy et al., 2013). However, not all the studies on the influence of CD36 are harmful, some demonstrate an anti-inflammatory effect. For example, the antidiabetic drug rosiglitazone activated microglial PPARγ and facilitated overexpression of microglia CD36 after ischemic stroke, which led to the sweeping away of peripheral immune cells infiltrated in the brain, benefiting the termination of the post-stroke neuroinflammation process (Garcia-Bonilla et al., 2015).

The TLR family is considered to be pivotal receptors mediating LD accumulation within microglia. TLRs are a critical innate immune receptor family involved in the inflammatory process evoked by microglial activation in stroke (Fang et al., 2013). TLR2, TLR4, TLR3 and TLR9 all play roles in ischemic cerebral injury (Ji et al., 2016; Tajalli-Nezhad et al., 2019). Cerebral ischemia induces the increase of TLR2 expression mainly in microglia. TLR2-deficiency inhibits microglial inflammatory activation, enhances the levels of synaptic remodeling markers and promotes survival rates of microglial (Gorup et al., 2019; Ma et al., 2020). However, there is no consensus about whether TLR2 blockade can alleviate the harm of ischemic stroke in mice (Hua et al., 2009; Bohacek et al., 2012; Zhao et al., 2017). TLR4 is mainly expressed by microglia in cerebral tissue (Jung et al., 2005). The increased expression of TLR4 activated the NF-κB pathway, released the pro-inflammatory cytokines TNF-α and IL-6, and exacerbated damage and apoptosis of neurons (Tajalli-Nezhad et al., 2019). Overexpression of TLR4 is an upstream sensor of post-ischemia inflammation and is related to poor outcome in stroke patients (Heneka et al., 2014). The level of serum TLR4 was a potential predictor of poor clinical outcome at 12 months after intracranial hemorrhage (Lei et al., 2024). In the human cortex cell culture, TLR4 inhibition reduced the activation of microglia pro-inflammatory phenotype and ROS production induced by lipopolysaccharide (LPS) (Parada et al., 2019). In TLR4-inhibited post-ischemia model, the infarct volume was reduced and the neuromotor function was improved (Gong et al., 2023). The activation of TLR4 combined with phagocytosis of myelin and cellular debris might lead to the formation of LDs in microglia. TLR4 signaling pathway activation is initiated by binding its extracellular domain to exogenous or endogenous ligands. LPS is a TLR4 ligand and stimulates the production of LDs both *in vivo* and *in vitro* (Burguillos et al., 2015; den Brok et al., 2018). It has been found that ApoA1 binding protein could bind to activated microglia via the binding site of TLR4, reducing LD accumulation in microglia in the spinal cord and generating anti-inflammatory influence in a peripheral neuropathy model (Navia-Pelaez et al., 2021). In a word, the activation of TLR4 in microglia participates in the formation of LDs.

TREM2 is an innate immune receptor of the TREM family which is uniquely expressed by microglia in the brain. It is a transmembrane protein responsible for lipid transport and microglia activation (Wu et al., 2017; Zhai et al., 2017; Deczkowska et al., 2018; Lewcock et al., 2020). It routinely perceives and increases LDL uptake in the healthy brain, and mediates microglial lipid sensing by binding with lipid ligands (Yeh et al., 2016; Lessard et al., 2018). TREM2 can greatly ease a variety of lipid burdens in the brain, including cholesteryl esters, diacylglycerol and triacylglycerol, and alter the state of microglia from physiological to DAM (Li et al., 2022c). The disruption way of the dynamic balance of lipid metabolism induced by TREM2 deficiency presents different depending on disease patterns. TREM2 knockdown microglia present like the foamy microglia in chronic inflammatory diseases. They exhibit severe cholesteryl esters storage impairment, resulting in the overloading of a variety of lipids within microglia and producing peroxidation-activated pro-inflammatory toxic (Do et al., 2022; Li et al., 2022c). However, TREM2-null mice failed to form LDs rich microglia in cerebral ischemic lesions (Walther et al., 2017). TREM2-deficient mice show a significant decrease in LDs accumulating microglia and worse neurological function after MCAO and reperfusion (Claes et al., 2021; Awuah et al., 2025). In stroke models, the increased quantity of microglia after ischemia stroke was suppressed in TREM2-null models, and the proliferation and phagocytosis ability of microglia were inhibited, therefore causing neural function deterioration (Wang et al., 2023; Wei et al., 2024; Awuah et al., 2025). TREM2 activation attenuates neuroinflammation and exerts a protective force in post-stroke damage, including ischemic and hemorrhage (Chen et al., 2020b). TREM2 upregulation may help promote the M2 anti-inflammatory microglial phenotype and reduce post-ischemic inflammation and stroke pathology progression by influencing lipid metabolism (Zhang et al., 2019; Wei et al., 2024). TREM2 does not have an intrinsic signaling pathway and its activation leads to the membrane adaptor protein-DNAX-activating protein of 12 kDa and extracellular signal-regulated kinase phosphorylation, modulating lipid metabolism, immune responses, proliferation and phagocytic capacity (Gouna et al., 2021). TREM2 suppresses stroke triggered inflammation by regulating the TGF-β1/Smad2/3 signaling pathway. TREM2 overexpressed microglia demonstrate increased ability of phagocytosis and decreased gene transcription of TNF-α, IL-1β, and NOS. Deficiency of TREM2 in microglia causes increased gene transcription of TNF-α, IL-1β, and NOS (Takahashi et al., 2005). However, there is opposite evidence proving that TREM2 impedes microglia-mediated hematoma and cell debride clearance, and impairs functional recovery of intracerebral hemorrhage (Zhou et al., 2023).

### Lipid droplets in microglial phagocytosis, proliferation, and pro-inflammation

It has been reported that the accumulation of LDs in microglia is negatively correlated with its phagocytosis. Inhibition of LD formation in microglia can restore its phagocytic function in AD. Human fat storage-inducing transmembrane protein 2 is a key protein in the biosynthesis of LDs which is significantly increased in AD mice. The absence of transmembrane protein 2 not only significantly reduced the TG level in the brain microglia of AD mice, but also enhanced the phagocytosis ability of microglia (Wu et al., 2025).

Microglia must endure profound metabolic conversion to meet the high energy demands charged by the phenotypic changes and morphologic transformations, to sustain phagocytosis and proliferation ability. Microglia receive multiple signals after ischemia and induce different metabolisms through a developing process within different subsets of microglial exhibiting various phenotypes (Beuker et al., 2022). After ischemic stroke, a number of factors can evoke LD synthesis in microglia, including inflammation context and the phagocytosis of cell debris. Both LPS and hypoxia inflammation intervention could mimic this environment *in vitro*. The pro-inflammatory shift triggered by LPS induces the accumulation of LDs in cultured microglia cells (Khatchadourian et al., 2012). Hypoxia-induced metabolic activation of microglia is pro-inflammatory, which involves lactate producing, glycolysis enhancing, the pentose phosphate pathway activating, and mitochondrial oxidative phosphorylation harming (Ghosh et al., 2018). The pentose phosphate pathway and aerobic glycolysis favor microglia lipid synthesis essential for anabolic courses, like microglia proliferation (Vander Heiden et al., 2009; O’Neill et al., 2016). Proliferating microglia are shifted to a pro-repair state capable of releasing protective factors, such as IGF-1 (Zhao et al., 2017; Planas, 2024).

In the TLR4 signal pathway activation, the first step is TLR4 recruitment into lipid rafts, then the second is the formation of the TLR4/myeloid differentiation protein-2 (MD2) complex, activating downstream signaling cascades (Fu et al., 2014; Gianfrancesco et al., 2018). Lipid rafts are membrane microdomains composed of glycolipid, sphingolipid and cholesterol. They are considered as platforms that contribute to the interaction of the TLR4/MD2 complex, regulating signal transduction (Allen et al., 2007). The key enzyme for the synthesis of sphingomyelin, sphingomyelin synthase 2 has critical roles in manipulating membrane fluidity and lipid rafts’ structure. Sphingomyelin synthase 2 deficiency generates an anti-inflammatory action by decreasing the TLR4/MD2 complex in microglia and inhibiting enrollment of TLR4 into lipid raft thereby ameliorating cerebral ischemia-reperfusion injury (Xue et al., 2019). The recruitment of TLR4 into lipid rafts can be induced by cerebral ischemia-reperfusion. Pro-inflammatory microglia are activated and poor outcomes of ischemic stroke are induced by TLR4 signal pathway (Jung et al., 2005; Powers et al., 2006).

LD has been considered a temporary container of neutral lipid, manipulating the spatiotemporally concerted release of lipid inflammatory mediators (Haemmerle et al., 2011). These released lipid mediators could control the NF-κB signal pathway and modulate the inflammation gene expression (Ong et al., 2011; Jarc and Petan, 2020). NF-κB activation in microglia propels neuronal ischemic injury (Chen et al., 2024). This procedure is triggered by an inflammatory multiprotein signaling complex, inflammasome. Inflammasome is activated when cells perceive danger, and its duty is sending signals to activate the immune system by transmitting particular cytokines (Xu et al., 2024). Inflammasome-mediated production of IL-1β is deployed following activation of NF-κB at the transcriptional level and then the inactive pro-form has to be sundered for activation by caspase-1 (Dinarello, 2009). Activation of caspase-1 can induce a quickly pro-inflammatory way of cell death named pyroptosis (Tsuchiya, 2020). Several studies reported that suppressing caspase-1 could protect ischemic brain by decreasing infarct size and attenuating neurological dysfunction (Pan et al., 2022; Ye et al., 2022; Wang et al., 2024). It has been pointed out that this protection is facilitated by suppressing NF-κB activation and the immune reaction of microglia (Li et al., 2019).

IFN response process also contributes to ischemia stroke-induced LD biosynthesis in microglia (Arbaizar-Rovirosa et al., 2023). Cerebral ischemia causes inflammation and cell death, enhancing the demand of phagocytosis to clear away dead cells, necrosis tissue, myelin debris, and infiltrated immune cells (Otxoa-de-Amezaga et al., 2019). During this course, it is shown that microglia exert an obvious type I IFN signaling response seemingly attributable to the sensation of threats through the cGAS/Sting pathway, therefore upregulating the releasing of LD-related innate immune molecules (Li et al., 2020a; Liao et al., 2020). Ischemia-induced microglial mRNA upregulate IFN-induced molecules associated to LDs (Arbaizar-Rovirosa et al., 2023). Moreover, the IFN signaling pathway related to ischemia-induced LD biogenesis is inhibited in Stat1 knockout mice deficient in IFN signal transduction (Arbaizar-Rovirosa et al., 2023). The IFN response process after ischemia is even stronger in the microglia of old mice compared to young ones, but the difference is eased after repopulating the microglia of old mice, consistent with the level of LDs (Arbaizar-Rovirosa et al., 2023).

## Coordinative Function of Peripheral Lipid Metabolism and Microglia in Stroke

Diseases including metabolic disorders and obesity, featured by major shifts in lipid metabolism, demonstrate activation of microglial inflammation (Gao et al., 2017; Cope et al., 2018). It was proven by several studies that high-fat diet (HFD) changes brain inflammatory environment (Maldonado-Ruiz et al., 2017), in a microglia-dependent (Valdearcos et al., 2017; Cope et al., 2018; Sharma, 2021; Ullah et al., 2021), but body weight-independent way (Sharma, 2021; Wang and Li, 2021). The microglia/macrophage increase was perceived in the brains of prolonged HFD-induced obese models after MCAO. The modified inflammatory response in HFD models may lead to a more serious neurological impairment after stroke (Maysami et al., 2015). In the brain, and particularly the brainstem, TGF-β-activated kinase 1 in microglia takes part in the formation of prolonged obesity-induced cerebrovascular abnormalities (Shen et al., 2020). One clinical trial has perceived that hyperlipidemia is associated with poorer neurological function of stroke patients, while administrating cholesterol restriction drugs can changeover this adverse outcome because of their pleiotropic impacts on neuroinflammation, oxidative stress and vascular integrity (Restrepo et al., 2009). In the following context, we summarize the current knowledge on the unique roles of several peripheral lipids, their derivatives and lipases on microglial cells and inflammation after stroke (**Table 1**).

**Table 1:** Function of peripheral lipid metabolism in cerebral ischemia

Lipid/lipase/lipid kinase	Subtype	Function	Reference
DHA/EPA		Diminishes oxidative stress injury; Attenuates microglial activation and inflammation; Regulates phagocytic response of microglial	Orr et al., 2013; Corsi et al., 2015; Fourrier et al., 2017; Inoue et al., 2017; Leyrolle et al., 2019
Prostaglandin E2			
	EP1	Promotes ischemic brain injury; EP1 antagonists or EP1 deficiency reduces expression of neurotoxic factors, and rescues brain tissue and infarct volume	Saleem et al., 2007; Frankowski et al., 2015; Mushtaq, 2023
	EP2	Controversial; Lack of EP2 signaling reduces the number of infiltrating immune cells and infarct size; EP2 receptor activation is neuroprotective against excitotoxic and anoxic injury; EP2 knocking out significantly increases infarct size	McCullough et al., 2004; Liu et al., 2019a
Lipoxin A4		Anti-inflammatory; Alleviates neurological dysfunction and attenuates neuronal apoptosis after cerebral ischemia	Sobrado et al., 2009; Ye et al., 2010; Chi et al., 2023
Resolvin		Promotes the resolution of neuroinflammation and decreases microglial activation	Rey et al., 2016; Roohbakhsh et al., 2022
	Resolvin D1	Reduces ischemic core size and penumbra areas; Induces recovery of neural dysfunction; Promotes the phagocytosis of neutrophils by microglia; Alleviates neuroinflammation	Chen et al., 2020a; Qiu et al., 2021; Denorme et al., 2022; Li et al., 2022a, 2023b; Reid et al., 2023b
	Resolvin D2	Reduces neuroinflammation and improves neural function	Dong et al., 2019
Neuroprotectin		Reduces infarct volumes via inhibiting microglial and NF-κB inflammatory activation	Marcheselli et al., 2003; Bazan et al., 2012
Leukotriene		Regulates inflammation and exerts neurotoxic effect, resulting in poor clinical outcomes in ischemic stroke patients	Shi et al., 2015; Chan et al., 2020
ATGL		Inhibition of ATGL can stop the release of pro-inflammatory cytokines in microglia by reducing LDs lipolysis, can reduce infarct size and improve neurofunction as well	Li et al., 2023a; Robb et al., 2025
Sphk			
	Sphk1	Leads to systematic inflammation Exacerbates stroke outcomes	Spiegel and Milstien, 2011; Zheng et al., 2015
	Sphk2	Protective in experimental ischemic stroke	Pfeilschifter et al., 2011
PI3Kγ		Reinforces microglia phagocytosis and reduces brain infarction	Schmidt et al., 2013, 2016

ATGL: Adipose triglycerides lipase; DHA: docosahexaenoic acid; EPA: eicosapentaenoic acid; EP: prostaglandin E receptors; NF-κB: nuclear factor κB; PI3Kγ: phosphatidylinositol-3-kinase γ; Sphk: sphingosine kinases.

### Polyunsaturated fatty acids and their derivatives

A variety of lipid mediators like PUFAs and their metabolites produced after brain ischemia can change the inflammatory process and promote the regression process after brain ischemia (Reid et al., 2023a). Cerebral ischemia leads to the accumulation of PUFAs, such as arachidonic acid, eicosapentaenoic acid (EPA), and docosahexaenoic acid (DHA), all of which are derived from phospholipids that build cell membranes. PUFAs are further classified into three types: n-3, n-6, and n-9 fatty acids. Numerous studies to date have shown that n-3 and n-6 PUFAs, or their derivatives, modulate neuroinflammation and other pathologies of cerebral ischemia (Asatryan and Bazan, 2017; Gonzalo-Gobernado et al., 2019; Chumak et al., 2022). PUFAs have been considered as beneficial for ischemic stroke. Its derivatives also promote tissue repair by inhibiting excessive inflammatory reaction, reducing pro-inflammatory mediators and increasing anti-inflammatory cytokines (Miao et al., 2021). DHA and EPA reduce the production of nitric oxide, release pro-inflammatory factors and diminish oxidative stress injury in LPS-treated microglia (Corsi et al., 2015; Inoue et al., 2017). It has been confirmed that microglial activation and inflammation triggered by various pro-inflammatory treatments (HFD, and LPS) can be attenuated by DHA and EPA *in vivo* (Orr et al., 2013; Fourrier et al., 2017). The phagocytic response of microglial in a variety of diseases and phases could also be differentially regulated by PUFAs (Leyrolle et al., 2019).

Oxylipins are typical lipid mediators in acute inflammatory process and play important roles in the innate immune response, including n-6 PUFA-derived prostaglandins, lipoxins and n-3 PUFA-derived resolvins and neuroprotectin. Arachidonic acid is the most abundant n-6 PUFA in the body and can be oxidized to produce prostaglandins, lipoxin and leukotrienes. Prostaglandin E2 has been found to play a pivotal role in neurological diseases including stroke and several neurodegenerative diseases (Cimino et al., 2008; Sluter et al., 2023). It has four prostaglandin E receptors including EP1, EP2, EP3 and EP4. The regulation of neuroinflammation depends on which receptor in the brain cells is activated (Breyer et al., 2001; Kawano et al., 2006). The EP1 signaling pathway facilitates ischemic brain damage because EP1 antagonists or EP1 deficiency reduces the expression of intercellular adhesion molecule-1, MMPs and neurotoxic factors and decreases infarct size (Frankowski et al., 2015). Inhibition of EP1 activity rescues brain tissue in models of ischemic stroke (Saleem et al., 2007; Mushtaq, 2023), reduces microglial activation and weakens brain damage in mouse models of intracranial hemorrhage (Zhao et al., 2015). A report showed that the lack of EP2 signaling in myeloid and neuronal cells reduced the amount of infiltrating immune cells and infarct size (Liu et al., 2019a). However, it has also been reported that activation of the EP2 receptor is neuroprotective against excitotoxic and anoxic injury (McCullough et al., 2004). EP2 knocking out significantly enhanced infarct volume in transient forebrain ischemia model (McCullough et al., 2004).

Lipoxin A4 alleviates neurological dysfunction and attenuates neuronal apoptosis after ischemic stroke. The neuroprotective effect against ischemia injury might be related to its anti-inflammatory property (Chi et al., 2023). A lipoxin A4 analogue treatment blocked the activation of microglia and astrocytes, decreased the release of pro-inflammatory factors like TNF-α and IL-1β; and increased the expression of restorative cytokines IL-10 and TGF-β1 in the ischemic brain (Ye et al., 2010). The lipoxin A4 analogue also increased PPARγ transcriptional activity and its neuroprotection effect is partly suppressed by the PPARγ antagonist in experimental stroke (Sobrado et al., 2009).

Resolvins from the DHA- or EPA-derived promote the resolution of neuroinflammation and decrease microglial activation (Rey et al., 2016; Roohbakhsh et al., 2022). Resolvin D1 combined with neuroprotectin D1 might reduce ischemic core size and penumbra areas, and induce recovery of neural dysfunction (Reid et al., 2023b). Resolvin D1 also reprograms energy metabolism to facilitate the phagocytosis of neutrophils by microglia after cerebral ischemia (Li et al., 2023b). In this way, neutrophil accumulation is reduced and neuroinflammation is alleviated in the ischemic brain. Neutrophils enter the brain first and accelerate infiltration and accumulation of other immune cells thereby intensifying inflammation and brain damage after cerebral ischemia (Qiu et al., 2021; Denorme et al., 2022; Li et al., 2022a). In a rat MCAO/reperfusion model, intraperitoneal injection of resolvin D1 reduced infarct size, cerebral edema and pro-inflammatory cytokines (Chen et al., 2020a). By inducing macrophage/microglia M2 polarization, resolvin D2 inhibited inflammation, and ameliorated brain damage and neurological dysfunction in diabetes mellitus related ischemic stroke (Tang et al., 2022). In a mouse cerebral ischemia-reperfusion model, venous delivery of resolvin D2-loaded nanovesicles reduced neuroinflammation and improved neural function (Dong et al., 2019). The release of neuroprotectins reduced infarct volumes via inhibiting microglial and NF-κB inflammatory activation, decreasing cyclooxygenase 2 expression and infiltrating polymorphonuclear leukocytes (Marcheselli et al., 2003; Bazan et al., 2012). Leukotrienes regulate inflammation and exert neurotoxic effects via activation of cysteine leukotriene receptors, resulting poor clinical outcomes in patients of ischemic stroke (Shi et al., 2015; Chan et al., 2020).

### Lipases and lipid kinase

During lipolysis process, lipases are accountable for the hydrolysis of lipids, of which adipose TG lipase was the first and major rate-limiting enzyme. Atglistatin, the specific inhibitor of adipose TG lipase, is demonstrated to stop the release of pro-inflammatory cytokines in microglia by reducing LD lipolysis (Robb et al., 2025). After treatment of Atglistatin, mouse models of MCAO showed reduced infarct size and improved neurofunctional presentation at the acute phase (Liu et al., 2023a).

Sphingosine kinases (Sphks) are key enzymes accountable for synthesizing the lipid signal transducer sphingosine-1-phosphate, which play main roles in cell responses including cell growth and death (Neubauer and Pitson, 2013; Ayub et al., 2021). Via the second messenger sphingosine-1-phosphate, the induced expression of Sphk1 has been shown to exert its function of leading to systematic inflammation through the expression of many kinds of pro-inflammatory mediators (Spiegel and Milstien, 2011). The microglial activation of pro-inflammatory cytokine by ischemic neurons can be inhibited in Sphk1-deficient mice (Zheng et al., 2015). The activated microglia produced pro-inﬂammatory cytokines like IL-1β, TNF-α, IL-6, and NLRP3 inﬂammasome is reduced, while the neurotrophic factors usually related to restorative functions, brain-derived neurotrophic factor and glial derived neurotrophic factor (GDNF), are increased by the sphingosine-1-phosphate receptor agonist ﬁngolimod (Noda et al., 2013; Yao et al., 2019). Though expression of Sphk1 and pro-inflammatory factors which is induced in microglia following MCAO exacerbates stroke outcomes, Sphk2 was found to be protective in experimental ischemic stroke (Pfeilschifter et al., 2011). Phosphoinositide 3-kinase γ reinforces microglia phagocytosis (Schmidt et al., 2013). Brain infarction was more pronounced and activated phagocytosis was decreased in phosphoinositide 3-kinase γ-knockout mice compared to wild-type in ischemic brains (Schmidt et al., 2016).

## Diet and Gut Microbiome Regulate Microglial Inflammation

### Diet

Not only lipid metabolism in brain, but also various diet patterns can influence inflammation response after stroke. It has been pointed out that some kinds of food or nutrition are pro-inflammatory while some are anti-inflammatory. For example, processed meats, artificial sweeteners and refined sugars, saturated fats and high-fat dairy products always consist in pro-inflammatory diets (Malesza et al., 2021). Long-term intake of certain food ingredients is usually related to the risk of stroke. It also has an influence on brain damage and function deficits after stroke (Spence, 2019).

HFD-induced obesity exacerbates stroke impairment by influencing the distribution of neurons, microglia/macrophages and astrocytes within infarct region (Fifield et al., 2019). Moderate dietary protein restriction prevents ischemic brain against inflammation and oxidant response impairment (de Carvalho et al., 2019). Moderately protein-reduced diet can also reduce brain infarct size and promote neurological restoration, increase long-term neuronal survival and suppress microglia accumulation in transient MCAO models (Silva de Carvalho et al., 2022). Acute and persistent hyperglycemia after stroke in both normal and diabetic individuals significantly worsen outcomes (Augestad et al., 2022). In diabetes mellitus patients, resolution of inflammation is hampered after ischemic stroke, attributed to pro-inflammatory factors increase and M1 microglia converting (Tang et al., 2022).

Anti-inflammatory diets always mainly contain fruits and vegetables. Natural unprocessed grains and unsaturated fats, like olive oil and n-3 PUFAs, are also significant components in anti-inflammatory diets. Protein from legumes, fish and chicken are considered to be more protective from inflammation than red meat (Scheiber and Mank, 2025). Mediterranean-like diet is a typical example of anti-inflammatory diets. It can help diminish pro-inflammatory reaction and attenuate oxidative DNA damage. This diet style is proven to reduce brain damage and functional impairments following ischemic stroke (Romaus-Sanjurjo et al., 2024).

Long-term dietary intervention with high n-3 PUFAs (alpha-linolenic-acid) decreases brain damage and neurological dysfunction, reduces resident microglia and restrains their activation (Bonetti et al., 2021). n-3 PUFA plays an anti-inflammatory role by suppressing NF-κB activation and subsequent release of microglia inflammatory mediators (Zhang et al., 2010). Recently, increasing evidence shows that phenols in regular diet demonstrate neuroprotective function and curb microglial activation. Natural phenols were proven to inhibit pro-inflammation pathways in microglia activating, such as the NF-κB, mitogen-activated protein kinase (MAPK) and Janus kinase (JAK)-signal transducer and activator of transcription (STAT). These pathways are important in microglia-mediated inﬂammation which occurs in various neuropathy like brain injury, infection, stroke, autism and Parkinson’s disease. Polyphenols are commonly found in apples, berries, dark chocolate, and hazelnuts (Mazza et al., 2021). Quercetin is a type of ﬂavonoid phenol abundant in vegetables and fruits like onions, tomatoes, radish and caper leaves (Vidak et al., 2015). In models of ischemic and hemorrhagic stroke, quercetin signiﬁcantly inhibited inﬂammation and oxidative stress, reduced infarct size and neuronal cell death rate (Zhang et al., 2015). Quercetin possibly exerts anti-inﬂammatory effect by suppressing nitric oxide production and inflammatory factor released in activated microglia (Kao et al., 2010). Long-term intake of resveratrol, a phenolic compound naturally present in grapes, berry skins and several plants, kept the cerebrovascular system from disruption in recurrent ischemic stroke (Clark et al., 2012). Phenols selectively curbed the pro-inflammation impact of activated microglia without disturbing their phagocytosis *in vivo* (Rangarajan et al., 2016).

Every-other-day feeding, named intermittent fasting, has shown the potential of extending lifespan and enhancing health status of mammals (Kim et al., 2020a). It promotes neuronal protection ability through increasing adaptive stress response and inhibiting neural inflammation (Matsui et al., 2018; Dias et al., 2021). Intermittent fasting exerts a protective effect against brain damage by suppressing microglia activation-associated inflammation, reducing pro-inflammatory factors released during both the acute and chronic phases of intracranial hemorrhage (Dai et al., 2022). The energy restriction diet, intermittent fasting, can enhance sirtuin 6 expression (Carreira et al., 2018). Sirtuin 6 activation exerts anti-inﬂammatory impact in microglia of stroke models, playing a neuroprotective role (He et al., 2021). Energy restriction diet might protect against M1 microglia activation-induced impairment in cerebral ischemia via enhancing sirtuin 6 expression after stroke (Song et al., 2022).

### Gut microbiome

Recent studies highlight the bidirectional communication between the gastrointestinal system and the central nervous system, named the gut–brain axis, as a critical modulator of neuroinflammation (Singh et al., 2018; Liu et al., 2024c; Xie et al., 2024). Current evidence shows that the gut microbiota can regulate microglial functions through the gut-brain axis (Arya and Hu, 2018). After stroke, nearly half of patients encounter gut microbiota dysbiosis, gut dysmotility, gut hemorrhage and even gut-origin sepsis (Mathias et al., 2024). Cytokines released and metabolites produced by the microbiota can migrate across the gut barrier and reach the brain injury site and significantly affect immune cells including microglia, and astrocytes (Wang et al., 2021). Both gut microbes and mucosal cells influence the release of immune molecules that affect the central nervous system, including the anti-inflammation factors TGF-β and IL-10, the pro-inflammation factors IL-1 and IL-8 (Shi et al., 2017; Kayama et al., 2020). Second, they directly impact the development and stability of microglia. This is important for manipulating central nervous system development and maintenance, like neurogenesis and myelination in the adult brain. They also play a critical role in immune surveillance in the brain. They clear cellular debris and take part in information transmission (Abdel-Haq et al., 2019). Third, they trigger immune cells, including dendritic cells, neutrophils and microglia. These cells then generate cytokines that promote inflammation, such as IL-1α, IL-1β, TNF-α, and IL-6. These cytokines migrate across the BBB and impact brain function (Camara-Lemarroy et al., 2020). Fourth, the gut microbiota influences the migration of certain immune cells in the intestinal tract to the brain. These cells have a critical role in exacerbating inflammatory process (Wastyk et al., 2021). In the face of an attack, abundant intestinal IgA-positive plasma cells translate to the central nervous system to mitigate neuroinflammation (Pröbstel et al., 2020). Lymphocytes and γδ T cells in small intestine move to the meninges and brain after stroke, worsening ischemic damage (Benakis et al., 2020).

After cerebral ischemia, brain injury leads to autonomic nervous system-derived autonomic norepinephrine release, reducing the number of intestinal epithelium cells, disrupting the function of mucus secretion and changing gut microbial communities into dysbiosis (Tan et al., 2021). The catecholaminergic stress response in the gut can be triggered by an ischemic stroke, leading to repeated gut dysbiosis, intestinal motility disturbance and intestinal barrier dysfunction (Iadecola et al., 2020). Mice with deficiency of segmented filamentous bacteria had critically larger cerebral infarct volume as a result of regulatory T cell expansion being inhibited (Sadler et al., 2017). Decreased brain injury size was even found after fecal microbiota transplanting from naïve animals to the MCAO mice (Singh et al., 2016).

Microbes in the gut synthesize metabolites such as short-chain fatty acids (SCFAs), trimethylamine N-oxide and LPS enter the bloodstream and exert effect in neurons and microglia in the central nervous system. When homeostasis of the gut microbiota is disrupted, gut dysbiosis leads to an imbalance of the gut microbiome and a decrease in SCFA production (Yamashiro et al., 2021). SCFAs are reported to manipulate neuroinflammation and cerebral remodeling post-stroke (Sadler et al., 2020; Benakis and Liesz, 2022). However, fecal SCFA production is found to reduce acutely in stroke patients (Tan et al., 2021). Restoring the microbiome several days from a young post-ischemic stroke model is reported to attenuate inflammation and promote recovery in old animals, primarily via SCFAs (Lee et al., 2020). At 24 and 72 hours after MCAO, high levels of SCFAs, especially sodium butyrate, have been shown to decrease the size of infarction and therefore improve neuro-functional outcomes (Zhou et al., 2021). They also exert a modulation effect on microglial activation and the permeability of the BBB (Kasarello et al., 2023). SCFAs modulate inflammation, control glucose metabolic processes and microglial proliferation and activation by triggering G-protein-coupled receptors or inhibiting histone deacetylases, and influence BBB integrity as well (Yamashiro et al., 2021). In AD, long-term supplementation with SCFAs has beneficial effects on energy metabolism in the brain and suppresses the pro-inflammatory reaction of microglia (Giridharan et al., 2019; Sun et al., 2023). In cerebral ischemia, sodium butyrate downregulated TNF-α and NOS-2 and upregulated IL-10 release of microglia (Patnala et al., 2017).

In addition, a study showed that trimethylamine N-oxide production increase by the copper transporter gene intervention in microbes leading to larger infarct volume after stroke (Zhu et al., 2021). Suppressing microbial trimethylamine lyases by the choline analog 3,3-dimethyl-1-butanol is also demonstrated to reduce the risk of cardiometabolic disease, by decreasing gut-synthesized trimethylamine N-oxide which is produced by the microbial metabolism of foods that are rich in trimethylamine (Wang et al., 2015). That result might provide some potential relationship with cerebral vascular disease.

LPS is considered to be a main component of bacteria. It is always generated from the damaged gut barrier and migrates into the brain, then triggers microglia and sets off the inflammation process. LPS also stimulates the secretion of many inflammatory cytokines including IL-1β, TNF-α, and IL-6 in the brain through the upregulation of TLR4 and CD14 expression (Yang et al., 2020). In addition, these cytokines are mentioned to activate TLR4, creating a positive feedback loop (Zhao et al., 2021).

Gut microbiota is becoming one of the main targets of immune regulation research in stroke. Dysbiosis of the gut microbiota reflects changes in the constitution and function of the gut flora, which has been shown to be a potential risk factor for poor stroke prognosis and a potential therapeutic target for stroke. Moderate protein restriction diet after ischemic stroke induces sustained neurological recovery with gut microbiota rebalancing (Silva de Carvalho et al., 2022). Atorvastatin-induced anti-inflammatory function in stroke models involved restitution of gut microbiota, enhancement of gut barrier integrity and regulation of gut immunity (Zhang et al., 2021b). By preventing the pro-inflammatory polarization of microglia/macrophages, berberine markedly reduced neuroinflammation and ameliorated neurological dysfunction in intracranial hemorrhage mice. Berberine is a candidate treatment for intracranial hemorrhage-caused inflammation, with its effects at least partially dependent on gut flora (Liu et al., 2023a). Therapy alternatives such as fecal microbiota transplantation, a high-fiber diet and probiotic has been recommended, but all of them has not yet been studied in any clinical trial (El-Salhy et al., 2021; Zhong et al., 2021; Czerwińska-Rogowska et al., 2022).

## Therapeutic Strategies Targeting Lipids and Microglia After Stroke

Active cerebroprotection is essential for stroke treatment. The neuroinflammation is always an important therapeutic target. In recent years, a series of studies have been conducted globally on the field. Disruption of lipid metabolism and post-stroke neuroinflammation significantly influence stroke recovery. Microglia, essential for neuroinflammation, have significant interaction with lipid pathways, making the targeting lipid metabolism and microglial function a promising therapeutic strategy. Several novel therapeutic approaches, including pharmacological and nutritional interventions, have been proposed. For improving the outcome of stroke, treatments targeting lipids and microglia are promising.

### Potential drug interventions

#### Drugs targeting lipid metabolism

Lipids serve as critical mediators in energy supply and inflammation. Dysregulated lipid metabolism after stroke contributes to oxidative stress and exacerbates neuronal injury (Cerasuolo et al., 2024), which can significantly impact recovery and outcomes. Presently, drugs including statins and PCSK9 inhibitors (PCSK9i) are available to help regulate lipid metabolism. Statins are noted for their anti-inflammatory effects as well as their ability to lower lipid levels. A systematic review demonstrated that low-dose statin treatments significantly reduce high-sensitivity C-reactive protein and C-reactive protein concentrations (Alikiaii et al., 2021). By reducing inflammatory markers such as C-reactive protein, statins may help lower stroke risk, enhance endothelial function, decrease oxidative stress, and provide anti-inflammatory effects, which collectively contribute to their potential therapeutic benefits (Choudhary et al., 2023). Additionally, the antioxidant properties of statins are crucial in augmenting their neuroprotective effects (Morofuji et al., 2022). As ongoing research investigates the comprehensive range of statin effects, it becomes evident that these medications hold promise not only for cholesterol management but also for reducing the risk of stroke and related conditions. PCSK9i, which are a type of monoclonal antibody, have proven effective in significantly decreasing LDL cholesterol levels, with well-documented positive effects on cerebrovascular outcomes. Evidence from both preclinical and clinical research suggests that PCSK9i may decrease the inflammatory burden within atherosclerotic vessels, partially apart from their LDL cholesterol reduction effects (Barale et al., 2021; Marfella et al., 2023). Research suggests that PCSK9i possesses the ability to reverse pro-inflammatory responses and reduce systemic inflammation induced by LDL cholesterol in patients with familial hypercholesterolemia, and reduce cerebrovascular events (Marques et al., 2022). A prior investigation employing 18F-fluoro-deoxyglucose positron emission tomography/computed tomography to assess vascular wall inflammation *in vivo* indicated that PCSK9i may locally reduce low-grade inflammation within the arterial wall without influencing systemic inflammatory markers (Marques et al., 2022). From a mechanistic standpoint, various intermediate mediators, such as Lp(a), may provide insight into the inflammation-reducing effects of PCSK9i. Consequently, more research is required to uncover the potential mechanisms that explain the anti-inflammatory effects of PCSK9i.

#### Drugs regulating microglial function

Managing microglial polarization at various points in stroke can decrease neuroinflammation by promoting the M2 phenotype and blocking the M1 phenotype, thereby maintaining the post-stroke balance of anti-inflammatory and pro-inflammatory responses. The use of PPAR agonists, particularly those that activate PPARα, has been associated with reduced oxidative stress and inflammation, improving neurological outcomes in stroke models. A number of studies have revealed that PPARα activation can inhibit microglial overactivation and lower the expression of IL-1β and TNF-α in the hippocampus (Marques et al., 2022). Research on animal models of cerebral ischemia has revealed the neuroprotective benefits of PPARα agonists. Preclinical evidence indicates that these agonists can attenuate neuroinflammation and mitigate further brain damage following a stroke. Notably, an increase in infarct volume has been observed in PPARα knockout mice. However, evidence suggests that the benefits of PPARα activation may not be uniformly distributed across sexes, with males potentially deriving greater benefits than females (Park and Choi, 2017). Evidence from clinical research suggests that a PPARα and statin combination is effective in minimizing the risk of cardiovascular events and ischemic strokes in targeted patient populations (Kim et al., 2019a). PPARα agonists show significant promise as neuroprotective agents in stroke therapy post-onset. Additional studies are needed to investigate the activation of PPARα as a potential therapeutic target for protecting against ischemic stroke.

Although the impact of minocycline on improving National Institutes of Health Stroke Scale scores appears minimal, the mechanisms by which minocycline modulates microglial-associated neuroinflammation remain inadequately understood. These results suggest new potential pathways for treating ischemic stroke. Curcumin has been shown to influence the balance between M1 and M2 phenotypes, providing protection in ischemic stroke by preventing M1 polarization induced by LPS and IFN-γ, as well as microglial-mediated pro-inflammatory responses (Liu et al., 2017). α-Lipoic acid has been demonstrated to influence the M1/M2 polarization of microglia, lower pro-inflammatory cytokine expression, elevate anti-inflammatory cytokine levels, and block the NF-κB signaling pathway, thereby conferring neuroprotective effects (Wang et al., 2018). Glycine, a simple nonessential amino acid, is recognized for its neuroprotective properties (Serhan et al., 2020). It is an essential component of numerous proteins and acts as a major inhibitory neurotransmitter by binding to glycine receptors to inhibit postsynaptic neuronal activity during ischemic stroke (Liu et al., 2019b). Furthermore, research has demonstrated that glycine inhibits M1 polarization. Glycine transporter type 1 inhibition has been found to reduce the size of infarcts, improve neurological functions, and decrease the death of neuronal and microglial cells after ischemic stroke injury (Cavalcante et al., 2025). These findings collectively point to new strategies and techniques for ischemic stroke management.

These investigations propose novel therapeutic strategies and methods to address neuroinflammation associated with microglia in the setting of ischemic stroke. Pterostilbene has shown potential in reducing neuroinflammation and brain damage after intracerebral hemorrhage in mouse models, revealing a new regulatory connection involving pterostilbene and optic atrophy protein 1-mediated mitochondrial fusion (Wu et al., 2023). In the intracerebral hemorrhage mouse model, administering exosomes from human adipose-derived mesenchymal stem cells significantly boosted neurological function, lowered hemorrhagic volume, reduced neuronal apoptosis, and affected microglial polarization (Zhao and Li, 2024). Moreover, miR-342-3p is recognized as a possible regulator of formyl-peptide receptor 1, which plays a role in the neuroprotective action of human adipose-derived mesenchymal stem cells during intracranial hemorrhage. Additionally, lipid nanoparticles hold promise as lipid-based delivery systems for targeted drug delivery aimed at modulating microglial activity (Zhao and Li, 2024).

The initial immune cells to respond after a stroke are microglia, and lipid metabolism is a crucial and tightly regulated part of microglial immunometabolism. Growing evidence suggests that drugs targeting lipid metabolism and microglial function are emerging as novel therapeutic options after a stroke.

### Nutritional interventions

#### Lipid metabolism–targeted interventions

Approximately 50% of the brain’s dry weight consists of lipids, including many phospholipids and fatty acids available from the diet. Therefore, nutritional interventions are a vital way to regulate neuroinflammation. Research indicates that n-3 fatty acids, such as EPA and DHA, can lessen neuroinflammation, assist in repairing membranes, and cause microglia to shift to an anti-inflammatory (M2) phenotype (Djuricic and Calder, 2024). Research indicates that diminished tissue and plasma levels of specialized pro-resolving mediators are associated with the advancement of atherosclerotic cardiovascular disease (Radbakhsh et al., 2022). In animal models, n-3 fatty acids are essential substrates for specialized pro-resolving mediators, which can increase their production (Calder, 2020a), lessen neuroinflammation (Calder, 2015, 2020b) and prevent the advancement of atherosclerotic plaques (Matsumoto et al., 2008). Moreover, research on animals has shown that EPA/DHA can reduce microglial inflammation by triggering silent information regulator factor 2-related enzyme 1 pathways (Matsumoto et al., 2008). Moreover, research on animals has shown that EPA/DHA can reduce microglial inflammation by triggering silent information regulator factor 2-related enzyme 1 pathways (Inoue et al., 2017). EPA/DHA, either alone or in conjunction with statins, may attenuate atherosclerosis according to animal model findings (Salazar et al., 2022). Additionally, reducing the intake of saturated and trans fats is beneficial for cerebrovascular health, as high consumption of these fats exacerbates systemic inflammation and LDL oxidation, thereby impairing cerebrovascular health (Sellem et al., 2023). Lipid-lowering nutrient methods, such as plant sterols can reduce LDL cholesterol by blocking intestinal absorption, and soluble fiber binds bile acids, lowering LDL (Cicero et al., 2023).

#### Microglia-targeted interventions

After stroke, microglia cells in the penumbra produce significant amounts of superoxide anions, hydrogen peroxide, and hydroxyl radicals. Oxidative stress plays an important cause of poor outcomes in stroke. The nutrients regulating microglia and antioxidative may be effective intervention measures. Polyphenols and vitamin D have properties that reduce inflammation and act as antioxidants. As a type of polyphenol, curcumin has been found to improve the inflammatory microenvironment and increase the neuroprotective potential of human umbilical cord mesenchymal stem cell transplantation through the mediation of anti-inflammatory microglia (Li et al., 2023c). In a rat model, curcumin-loaded gelatin nanoparticles significantly reduced neuroinflammation and neuronal apoptosis following a stroke (Yang et al., 2024). Curcumin reduces white matter damage caused by stroke and enhances functional recovery by preventing microglia pyroptosis through NF-κB suppression (Ran et al., 2021). Resveratrol, a stilbene, can reduce oxidative stress and microglial activation linking to improved cardiovascular health (Rana et al., 2022). Vitamin D regulates microglial activation and reduces pro-inflammatory cytokines to restrain neuroinflammation and brain injury in animal stroke models (Cui et al., 2023). Moreover, vitamin E, vitamin C, and selenium help fight oxidative stress associated with excessive microglial activation (Jabeen et al., 2023; Liang et al., 2023; Młynarska et al., 2024). Dietary potential to elicit therapeutic effects might be attributed, at least in part, to a bidirectional association with the gut microbiome. The therapeutic potential of dietary interventions may be partially attributed to their bidirectional relationship with the gut microbiome. Probiotics and prebiotic fiber have been shown to regulate post-stroke gut dysbiosis (Raghani et al., 2024), and reduce systemic LPS levels, which are known to drive microglial activation. Moreover, following a Mediterranean diet, which includes high levels of n-3 fatty acids, polyphenols, and fiber, while limiting saturated fats, has been linked to decreased inflammation after a stroke and better lipid profiles (Bayes et al., 2023).

Nutritional interventions to modulate neuroinflammation in stroke patients are still in their early stages, but a growing number of preclinical and clinical studies are providing evidence that this therapeutic approach is promising.

### Physical exercise

In rat models of transient MCAO, physical exercise provides neuroprotective benefits by influencing microglial activity, enhancing remyelination, and aiding in the restoration of neurological function (Xu et al., 2023). Additionally, physical exercise has the potential to regulate microglial activity, thereby mediating neuroinflammatory processes and contributing to the maintenance of brain homeostasis across various animal models (Strohm and Majewska, 2024). Physical exercise also enhances lipid metabolism in the brain and systemic circulation, creating a neuroprotective environment (Martemucci et al., 2024).

### Non-invasive neuromodulation

The field of stroke recovery is seeing non-invasive neuromodulation therapy as a new and emerging area (Keser et al., 2023). Transcranial direct current stimulation (tDCS) and repetitive transcranial magnetic stimulation (rTMS) have demonstrated promising outcomes in numerous studies. Specifically, rTMS has been observed to indirectly influence microglial activation by enhancing neuroplasticity and diminishing neuroinflammation. Animal models suggest that rTMS might alleviate ischemic stroke injury by suppressing M1 microglia polarization (Hong et al., 2022). Additionally, rTMS has been shown to alleviate cerebral injury and enhance motor function by modulating microglial polarization in MCAO mice, consequently improving the local inflammatory setting and hindering neuronal pyroptosis initiated by inflammasomes (Luo et al., 2022). Similarly, tDCS has the capacity to modulate microglial phenotype and morphology, which correlates with motor recovery in a mouse model of brain ischemia (Cherchi et al., 2022). Importantly, applying cathodal tDCS from days 5 to 9 after ischemia speeds up functional recovery in mice, affects neurogenesis, and decreases microglial activation by lowering CD16/32 expression linked to M1 phenotype (Walter et al., 2022). Microglia play a pivotal effect on regulating brain inflammation, influencing neuroplasticity, and promoting stroke recovery. Consequently, non-invasive neuromodulation therapy represents a promising strategy for modulating microglial activity.

Investigating the interaction between microglia and lipid metabolism presents significant potential for improving recovery outcomes following a stroke. Pharmacological strategies that target lipid metabolism and microglial regulation, in conjunction with nutritional and physical therapies, provide a comprehensive approach to reducing post-stroke complications. However, further research is essential to refine these interventions and facilitate their integration into clinical practice. When clinical evidence is sufficiently robust, it should be actively integrated into medical practice through quality management, promoting more personalized treatment models to benefit a greater number of patients.

## Opportunities and Limitations of Targeting Lipid Metabolism and Microglial Modulation for Stroke Treatment

**Figure 3:**
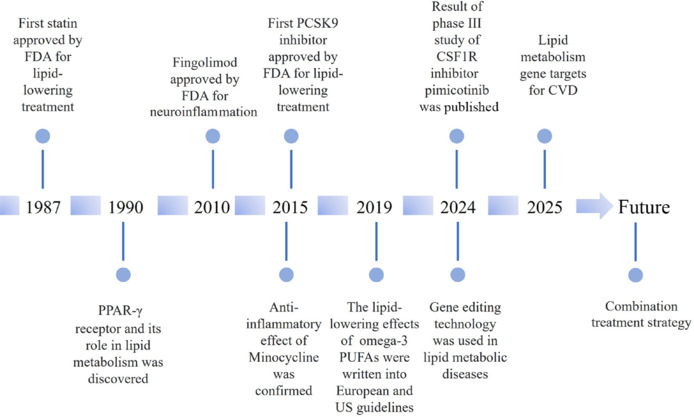
Milestones in stroke therapy with targeting lipid metabolism and microglia. The figure highlights key treatments aimed at lipid metabolism and microglial function. Statins and PCSK9 inhibitors received FDA approval in 1987 and 2015, respectively. The effects of PPAR and minocycline were later confirmed. Fingolimod was approved for neuroinflammation in 2010, and n-3 PUFAs were included in guidelines. CSF1R inhibitors show promise. Recently, lipid metabolism genes and gene editing technologies have been utilized. Combination treatments may be the future direction. CSF1R: Colony-stimulating factor 1 receptor; CVD: cardiovascular disease; FDA: U.S. Food and Drug Administration; PCSK9: proprotein convertase subtilisin/kexin type 9; PPAR: peroxisome proliferator-activated receptor; PUFA: polyunsaturated fatty acids.

The therapeutic targets associated with lipid metabolism and microglial modulation represents a hopeful direction for stroke therapy. Despite advancements in understanding the roles of lipid metabolism and microglial modulation in stroke, therapeutic development has been constrained. This section aims to explore therapeutic targets for post-stroke intervention, evaluate the U.S. Food and Drug Administration (FDA)-approved drugs and those undergoing clinical trials that target microglial and lipid-related pathways in stroke, and discuss the translational challenges and future strategies for drug development, with particular emphasis on personalized and combination therapies (**Figure 3**).

### Clinical trials targeting microglia and lipids in stroke

Fingolimod, also known as FTY720, modulates receptors for sphingosine-1-phosphate, and has demonstrated potential in preclinical stroke models by attenuating microglial activation and enhancing outcomes (Chamorro et al., 2016; Zhao et al., 2024). Additionally, it stabilizes lipid metabolism through the modulation of sphingolipid pathways. Fingolimod is presently undergoing evaluation in clinical trials (e.g., NCT02002390).

Similarly, minocycline, belonging to the tetracycline class of antibiotics, has been observed to curb microglial activation and diminish neuroinflammation in stroke models. It also mitigates lipid peroxidation and oxidative stress. Research conducted before clinical trials suggests that minocycline reduces the size of infarcts and enhances functional recovery in stroke models (Malhotra et al., 2018; Rok et al., 2020). Although not FDA-approved, its treatment effect has been investigated in clinical trials for stroke (e.g., NCT01805895). Results from a randomized controlled trial (NCT05487417) on thrombectomy treatment involving minocycline are awaited.

TTPU, known as 1-(1-propionylpiperidin-4-yl)-3-(4-(trifluoromethoxy)phenyl)urea, is a specific inhibitor of soluble epoxide hydrolase that has shown potential in altering lipid metabolism and decreasing neuroinflammation in stroke models (Tian et al., 2021). By blocking soluble epoxide hydrolase, TTPU increases endogenous cyclooxygenase levels, which results in anti-inflammatory, neuroprotective, and metabolic regulation (Huang, 2023). Additionally, TTPU has been demonstrated to reduce cell death in brain tissue and safeguard the BBB after ischemic incidents (Zhang et al., 2020). TTPU helps protect the BBB from ischemic stroke in both *in vivo* and *in vitro* models by decreasing apoptosis and inflammation, as well as regulating tight junction proteins (Wan et al., 2019). Currently, TTPU is being evaluated in early-phase clinical trials.

PPARγ agonists, which are approved for the management of diabetes, have demonstrated potential in mitigating microglia-mediated inflammation and enhancing lipid metabolism (Strosznajder et al., 2021). These agents may also contribute to the reduction of recurrent strokes and the stabilization of carotid plaques (Liu and Wang, 2023). However, their application in stroke treatment remains experimental, with ongoing clinical trials assessing their effectiveness and security. Extensive randomized controlled trials are required to assess the effectiveness and safety of PPARγ agonists for preventing secondary strokes and vascular events in patients who have had a stroke or transient ischemic attack.

Colony-stimulating factor 1 receptor inhibitors, which target microglial proliferation and activation, have shown potential in protecting against ischemic injury. Specifically, PLX3397 has been observed to suppress neuroinflammation in geriatric mice undergoing MCAO (Li et al., 2024). Although early-phase trials indicate promise in reducing neuroinflammation, challenges remain in balancing efficacy with potential adverse effects, such as impaired immune responses.

Curcumin is widely consumed in China and India, even as one of the culinary ingredients in food. Curcumin inhibits microglia/macrophage polarization and inflammatory responses, reducing neuroinflammation and protecting brain tissue from ischemic injury (Bhat et al., 2019). Although the current published stroke guidelines do not explicitly mention curcumin, it is recommended as adjuvant therapy to relieve neuroinflammatory by Chinese and Indian traditional medicine (Aggarwal et al., 2007).

Gene therapy has emerged as a cutting-edge strategy to modulate lipid metabolism and microglial behavior. Clustered regularly interspaced short palindromic repeats-associated proteins 9 (CRISPR-Cas9), a gene-editing tool can correct mutations or upregulate protective enzymes involved in lipid metabolism, such as LPL or ApoE (Lecuyer et al., 2021). Viral vectors can deliver genes encoding IL-10 or other anti-inflammatory mediators to suppress neurotoxic microglial activation (Hyun et al., 2010). Nanoparticles hold promise for delivering gene therapy agents with high precision in stroke treatment (Ghasemi et al., 2020). Furthermore, the application of nanoparticles, including polyethylene glycol and polyamidoamine-based systems, may enhance both the efficacy and targeted delivery to stroke-affected areas (Santos et al., 2018; Alonso-Alonso et al., 2022). In conclusion, considering the brain’s restricted regenerative capacity and the essential role of genes in stroke complications, gene therapy is a feasible therapeutic strategy (Ghasemi et al., 2020). The application of suitable nanoparticles in delivery further assures the practicality and effectiveness of this strategy.

### Approved treatments related to lipid metabolism and microglial modulation

PCSK9i has demonstrated the ability to lower LDL-C levels, decrease the development of atherosclerotic plaques, and provide anti-inflammatory effects, thereby reducing stroke risk. Marfella et al. (2023) demonstrated that these inhibitors not only decrease LDL-C levels but also attenuate inflammation within atherosclerotic plaques, offering both anti-inflammatory and antithrombotic advantages. PCSK9i is associated with a positive change in the inflammatory conditions of human atherosclerotic plaques, which might happen partly independent of their ability to lower LDL-C (Barale et al., 2021). Marques et al. (2022) further confirmed that PCSK9i modulates immune responses and reduces inflammation in the arterial wall, thereby contributing to a reduction in stroke risk.

Statins are extensively employed in stroke prevention due to their ability to lower cholesterol levels and stabilize atherosclerotic plaques. Their primary mechanism involves the inhibition of 3-hydroxy-3-methylglutaryl-coenzyme A reductase, leading to reduced serum cholesterol, decreased LDL-C, and enhanced cholesterol clearance (Liu et al., 2024b). Emerging evidence indicates that statins possess pleiotropic effects beyond the reduction of LDL-C (Liu et al., 2024b). These effects encompass anti-inflammatory and antioxidant properties, as well as improvements in endothelial function, all of which contribute to a reduction in stroke incidence and improved recovery following stroke (Marques et al., 2022). Moreover, statins offer several pleiotropic advantages, including enhanced endothelial function, anti-inflammatory properties, decreased oxidative stress, anti-thrombotic effects, and stabilization of atherosclerotic plaques (Khatiwada and Hong, 2024). Statins are recommended for blood lipid management in stroke patients, emphasizing their anti-inflammatory and plaque-stabilizing effects by Chinese Stroke Society (Liu et al., 2023b).

PUFAs also contribute to the modulation of lipid metabolism and inflammatory responses, thereby reducing atherosclerosis and neuroinflammation, which in turn lowers stroke risk (Bae et al., 2023). The American Heart Association and the American Stroke Association recommend high-dose n-3 PUFAs for the prevention of recurring conditions in patients with heart diseases, suggesting potential benefits for stroke patients (Kleindorfer et al., 2021).

A Chinese expert consensus recommends rTMS to improve stroke function recovery (Chinese Society of ECT & Neurostimulation, 2018), with high-frequency and intermittent theta-burst stimulation. Chinese clinical management guideline for stroke rehabilitation also recommended Non-invasive neuromodulation therapy, such as tDCS and rTMS (Liu et al., 2023b). Mayo Clinic reviewed various non-invasive neuromodulation techniques investigated to enhance recovery in patients after stroke (Keser et al., 2023). They found tDCS/rTMS have shown promising results (Feng et al., 2023; Stockbridge et al., 2023). Recently, European specialists have reassessed these guidelines, offering Level A support (indicating clear effectiveness) for using low-frequency rTMS to enhance hand motor recovery following a stroke (Lefaucheur et al., 2020).

Additionally, antioxidants like vitamin E and selenium are acknowledged for their important function in mitigating oxidative stress, which is related to various health issues, including lipid dysregulation and glial cell dysfunction. Evidence suggests that low doses of vitamin E may help prevent the onset of certain chronic diseases. A meta-analysis has shown that consuming more vitamin E in the diet is linked to a lower risk of stroke (Cheng et al., 2018). However, the evidence for the prevention of stroke is contradicting, and further randomized controlled trials are needed (Hantikainen and Lagerros, 2023). Vitamin E is generally considered safe at recommended doses (15 mg/day for adults), but high doses (> 400 IU/day) may increase the risk of hemorrhagic stroke due to its anticoagulant effects. Among the Inuit in Canada, higher dietary and blood selenium levels are linked to a lower prevalence of stroke (Hu et al., 2017). Vitamin E and selenium, which are dietary antioxidants, may be associated with positive beneficial health outcomes (Myhrstad and Wolk, 2023). However, evidence is insufficient to support routine clinical use after stroke (Cheng et al., 2018; Hantikainen and Lagerros, 2023).

### Challenges and problems in lipid metabolism and microglial regulation therapy after stroke

#### Timing and patient heterogeneity

The dynamic nature of post-stroke pathology complicates the timing of intervention. Microglia are rapidly activated after stroke and exhibit distinct phenotypes at different stages (Jayaraj et al., 2019). The timing of interventions targeting lipid metabolism and microglial regulation is critical. Acute-phase interventions may mitigate neuronal damage by inhibiting lipid peroxidation and modulating microglial activation, while subacute and chronic-phase interventions may enhance neurorepair by promoting lipid metabolic reprogramming and microglial phenotypic switching (Stoll and Nieswandt, 2019). However, the therapeutic effects may vary significantly depending on the time window, necessitating further research to identify optimal treatment timing. Patient heterogeneity also is a major factor influencing stroke treatment outcomes. Age, sex, genetic background, and comorbidities (e.g., diabetes, hypertension, and hyperlipidemia) may alter treatment responses by affecting lipid metabolism and microglial function (Prinz et al., 2019). For example, elderly patients may exhibit more severe lipid dysregulation and microglial activation, while sex differences in hormone levels may lead to varied therapeutic responses (GBD 2019 Stroke Collaborators, 2021). Additionally, genetic polymorphisms may influence the expression of lipid metabolism-related genes and microglial function. Future studies should focus on the following areas: (1) exploring the mechanisms and optimal time windows for interventions at different stages; (2) creating individualized treatment plans that cater to the different types of patients; and (3) utilizing multi-omics technologies and artificial intelligence to predict treatment responses and optimize therapeutic regimens.

#### Blood–brain barrier penetration

The BBB remains a significant barrier to delivering drugs or gene therapies to the brain, even after stroke-induced disruption. Innovative approaches for enhanced technology transport include intranasal delivery exploiting trigeminal and olfactory pathways, as well as techniques like temporary BBB opening through focused ultrasound combination with microbubbles (Kulkarni et al., 2024; López-Aguirre et al., 2024; Peng et al., 2025). Cell-penetrating peptides can enhance the uptake of therapeutic agents by facilitating their transport across endothelial cells (Ayuso-Dolado et al., 2021), but scalability and safety require further validation. There are challenges in BBB penetration for stroke therapy. Many therapeutic molecules, especially large or hydrophilic compounds, cannot cross the intact or partially disrupted BBB. The BBB undergoes dynamic changes after stroke, with an initial phase of increased permeability followed by partial recovery. This temporal variability affects drug delivery efficiency. The off-target effects also should be considered. Systemic administration of drugs may lead to unintended effects on peripheral tissues.

#### Safety and side effects

Targeting lipid metabolism or microglial activity carries risks of systemic side effects, including immune suppression or metabolic disturbances. Modulating microglial activity can inadvertently affect the broader immune system, potentially leading to increased susceptibility to infections or autoimmune reactions (Zhang et al., 2024). Both lipid metabolism and microglial activity are closely linked to inflammatory processes (Prinz and Priller, 2014). Interventions might trigger systemic inflammation or, conversely, suppress necessary inflammatory responses, leading to poor wound healing or chronic inflammation (Zhang et al., 2016). Balancing efficacy with safety is critical for clinical translation. Since microglia are crucial for brain homeostasis, targeting their activity might result in unintended neurological side effects, including cognitive impairments, mood disorders, or exacerbation of neurodegenerative conditions (Salter and Stevens, 2017; Wolf et al., 2017). Metabolic, liver and kidney toxicity, and off-target effects also should be considered (Trefts et al., 2017; Claeys et al., 2023). The long-term effects of modulating lipid metabolism or microglial activity are not fully understood, and there could be delayed adverse effects that manifest years after the intervention.

In the future, there are many directions. Integrating lipid-targeting drugs with anti-inflammatory agents may provide synergistic effects, improving outcomes without excessive side effects. Advances in omics discovery and imaging techniques may allow precision medicine targeting of lipid metabolism and microglial function. Artificial intelligence can accelerate the identification of novel drug candidates and optimize delivery strategies for stroke therapy.

By addressing these limitations and leveraging cutting-edge technologies, lipid metabolism and microglial modulation hold significant potential as therapeutic strategies for stroke recovery. Collaborations between researchers, clinicians, and industry stakeholders are vital for turning these promising concepts into clinical applications.

## Conclusion

We must acknowledge that we are still far from a profound understanding of the mechanism of inflammation and immune responses in stroke. The severity, the affected brain regions, the treatment, the pathological evolution, and the outcome are all different. Therefore, the role of microglia and lipid metabolism in inflammation after stroke is a double-edged sword, which is not only a key factor in brain damage after stroke, but also can inhibit inflammation to repair the damaged brain. Accumulating evidence suggests that microglia and lipid in neuroinflammation play a key role in the pathogenesis of ischemic stroke and have become an interesting target for therapeutic intervention. We need to better understand the involvement of inflammation and immune system in stroke, utilizing new technologies such as genomics and lipidomics, in order to achieve the final aim of modulating the inflammation system and innovating the treatment strategy. The limitations of this review are that more real-world studies need to be cited to elucidate the exact mechanisms of lipid metabolism and microglia in stroke. Several recent potential researches have shown that lipid metabolism and microglial modulation courses may be crucial therapeutic targets for stroke treatment in cells and animal models. Whether these discoveries in cell levels or animal disease models can be applied to humans in clinical trials or the real world is yet to be answered.

**Additional file:** *Open peer review report 1.*

OPEN PEER REVIEW REPORT 1

## Data Availability

*The data are available from the corresponding author on reasonable request*.
