# Supplementary material for: Lipid metabolism, microglia, and stroke
Source: Neural Regen Res. 2025 Aug 13;21(7):2779–95. doi: 10.4103/NRR.NRR-D-24-01523 (PMC13378828; doi:10.4103/NRR.NRR-D-24-01523)
Supplement: Supplementary file 1 [file NRR-21-2779_Suppl1.pdf]

OPEN PEER REVIEW REPORT 1

Name of journal: Neural Regeneration Research
Manuscript NO: NRR-D-24-01523
Title: Lipid Metabolism and Microglia Involved in Stroke
Reviewer's Name: Angel Céspedes
Reviewer's country: Colombia

COMMENTS TO AUTHORS

Overall evaluation on article quality:
Overall, it is a wide-ranging review of the intestinal biotic function interaction and lipid metabolism, from the close metabolic regulation between the gut-brain axis and microglial activity in the process of inflammatory process, to the benefit of reducing the risk for occurrence of cerebral infarction. I believe that this review covers some of the factors implicated in the pathogenesis of cerebral infarction and lipid metabolism's role in the processes of brain inflation with a clear focus on modulating the microglial cell system in a clear, deep and detailed way. This cellular metabolic interaction deserves to be published and shared scientifically.

Timeliness evaluation on article:
The given article confirms the consistency and completeness in searching for appropriate and recent information regarding the topic of interaction of intestinal biota with lipid metabolism and brain regulatory function concerning the main factors associated with inflammatory disease, namely with the preservation of the homeostatic balance of brain cells, energy metabolism and signal cascade pathways both in intact brain and in neuroinflammation and clarification of the relationship between lipid metabolism disorders and stroke pathogenesis. The authors establish the importance of considering the genes for lipid metabolism and their interaction with stroke and microglial function and phenotype evolution during the inflammation process on the heels of cerebral infarction.

Scope evaluation on article:
In connection with the analytical methodology provided by the authors to the subject of the review, and even assuming the references are more than sufficient for interpreting other authors information, the lack of interpretation at a deeper level concerning the exploration of novel pharmacological therapeutic targets, nutritional supplements and substitutes for lipid metabolism and microglial modulation after stroke was missing.

Direction evaluation on article:
The review article is very extensive and discusses in length the most recent findings of the scientific literature on the topic, revealing new avenues for scientific progress in the area of neuroinflammation and the intricate relationship with lipid metabolism.

Novelty evaluation on article:
Apparently, the article introduces quite new scientific concepts related to the role of the gut-brain axis, lipid metabolism and modulation of different cell lineages, particularly microglia cells in cerebral

infarction and other neuroinflammatory conditions.
